# Comparison of visual assessment and quantitative goodness-of-fit metrics on GUTS model fits

**DOI:** 10.1093/etojnl/vgae015

**Published:** 2025-01-06

**Authors:** Barbara Bauer, Alexander Singer, Oliver Jakoby, Dirk Nickisch, Thomas Preuss, Johannes Witt, Torben Wittwer, André Gergs

**Affiliations:** RIFCON GmbH, Hirschberg, Germany; RIFCON GmbH, Hirschberg, Germany; RIFCON GmbH, Hirschberg, Germany; RIFCON GmbH, Hirschberg, Germany; Crop Science Division, Bayer AG, Monheim, Germany; Crop Science Division, Bayer AG, Monheim, Germany; RIFCON GmbH, Hirschberg, Germany; Crop Science Division, Bayer AG, Monheim, Germany

**Keywords:** stakeholder survey, prediction quality, time resolved effect data, toxicokinetic-toxicodynamic (TKTD), model acceptance

## Abstract

For the application of toxicokinetic-toxicodynamic (TKTD) models in the European environmental risk assessment (ERA) of plant protection products, it is recommended to evaluate model predictions of the calibration as well as the independent validation data set based on qualitative criteria (visual assessment) and quantitative goodness-of-fit (GoF) metrics. The aims of this study were to identify whether quantitative criteria coincide with human visual perception of model performance and which evaluator characteristics influence their perception. In an anonymous online survey, > 70 calibration and validation general unified threshold models of survival (GUTS) fits were ranked by 64 volunteers with a professional interest in ecotoxicology and TKTD modeling. Participants were asked to score model fits to the time resolved survival data from toxicity experiments and to an aggregated dose-response curve representation. Dose-response curve plots tended to be scored better than time series, although both representations were based on the same toxicity test data and model results. For the time series, quantitative indices and visual assessments generally agreed on model performance. However, rankings varied with individual perceptions of the participants. Visual assessment scores were best predicted using a combination of GoF metrics. From the survey participants’ majority agreement on fit acceptance, GoF cut-off criteria could be derived that indicated sufficient fit performance. The most conservative GoF criterion well resembled current suggestions by the European Food Safety Authority. Hence, the survey results provide evidence that current quantitative GUTS assessment practice in ERA is consistent with perceptions of fit quality based on visual judgements of the dynamic model behavior by a large number of practitioners. Thus, our study fosters trust in model performance assessment.

## Introduction

Industrial agricultural practices heavily depend on the use of plant protection products (Oerke, 2006). Modern products are highly effective against pest organisms but also carry risks to nontarget organisms in the environment. Therefore, their regulatory approval for use in the European Union requires an environmental risk assessment (ERA, European Council, 2009). Current ERA procedures involve highly standardized laboratory tests to identify critical exposure concentrations. One approach to find the critical concentrations is to generate dose-response curves that link lethality at the end of the experiment to exposure. The dose or concentration at which 50% of the exposed organisms die (LD50 or LC50, European Food Safety Authority Plant Protection Products and their Residues [EFSA PPR], 2013; Stark et al., 2015) can be derived from these curves. The LD50 or LC50 depend on the exposure profile (e.g., constant or pulsed) and experiment duration. Therefore, when extrapolating to realistic, temporally variable exposure scenarios in the field, their relevance is limited (Ashauer et al., 2013; Jager et al., 2006).

The dynamics of toxic effects caused by temporally variable exposure to a substance can be addressed with mechanistic modeling approaches (Jager et al., 2006; Kooijman & Bedaux, 1996). Toxicokinetic-toxicodynamic (TKTD) models represent the processes influencing internal exposure of an organism (TK) and those that link internal exposure to damage and observable endpoints such as mortality (TD). The general unified threshold model of survival (GUTS; Jager et al., 2011) is a TKTD modeling framework with standardized equations, but substance- and species-specific parameter values (Jager & Ashauer, 2018a). A parametrized GUTS can be used to predict the potential risks of lethal effects from time-variable or constant exposure profiles in aquatic ERAs (Brock et al., 2021). In the absence of data on internal concentrations, a reduced model (GUTS-RED) is used. General unified threshold model of survival-RED can be parameterized (calibrated) based on single-species toxicity test data using one of several already existing computational implementations (e.g., openGUTS, Jager, 2019; morse R package, Baudrot & Charles, 2021; GUTS R package, Albert et al., 2022). The performance of the parametrized model should be evaluated by comparing model predictions with experimental data that were not used for calibrating the model (i.e., independent measurements), a procedure usually referred to as model validation (EFSA PPR et al., 2018).

To evaluate model performance, the fits to both the calibration data and the validation data are assessed using a combination of visual assessment and quantitative criteria (Focks et al., 2018). The procedure is recommended for TKTD model applications in the European ERA (EFSA PPR et al., 2018). A visual assessment is essential to check whether the model predictions follow a plausible time-course. Furthermore, the visual representation of the fit can reveal reasons that are underlying potential mismatches (e.g. Singer et al., 2023). In contrast, quantitative goodness-of-fit (GoF) metrics do not inform about the time course of model dynamics but provide a reproducible and objective way to characterize model performance. Three criteria were suggested by EFSA PPR et al. (2018) for the aquatic risk assessment: one that takes into account the uncertainty in the model predictions (posterior predictive check [PPC]), one that measures the match over time (normalized root-mean-square-error [NRMSE]), and one that considers the final match between model prediction and data (survival probability prediction error [SPPE]). The metrics were originally suggested to judge the acceptability of validations, but they can also be used to assess the calibration fit. Although metrics may play an important role in the evaluation of model acceptability, detailed guidance for ERA on their interpretation has not been published. This is mostly due to the lack of examples applying the metrics to TKTD model results (but for recent developments on GUTS, see Bauer et al., 2024; Focks et al., 2018; Nickisch Born Gericke et al., 2022; Singer et al., 2023 and on dynamic energy budget-TKTD, see Koch et al., 2024; Martin et al., 2024) such that future experience with applications of TKTD models might lead to adaptation of the metrics and acceptability criteria (EFSA PPR et al., 2018). Comparing metric values and qualitative (visual) assessments for several fits can help to gain an insight into their complementarity, redundancy, and eventual contradictions.

**Figure 1.**
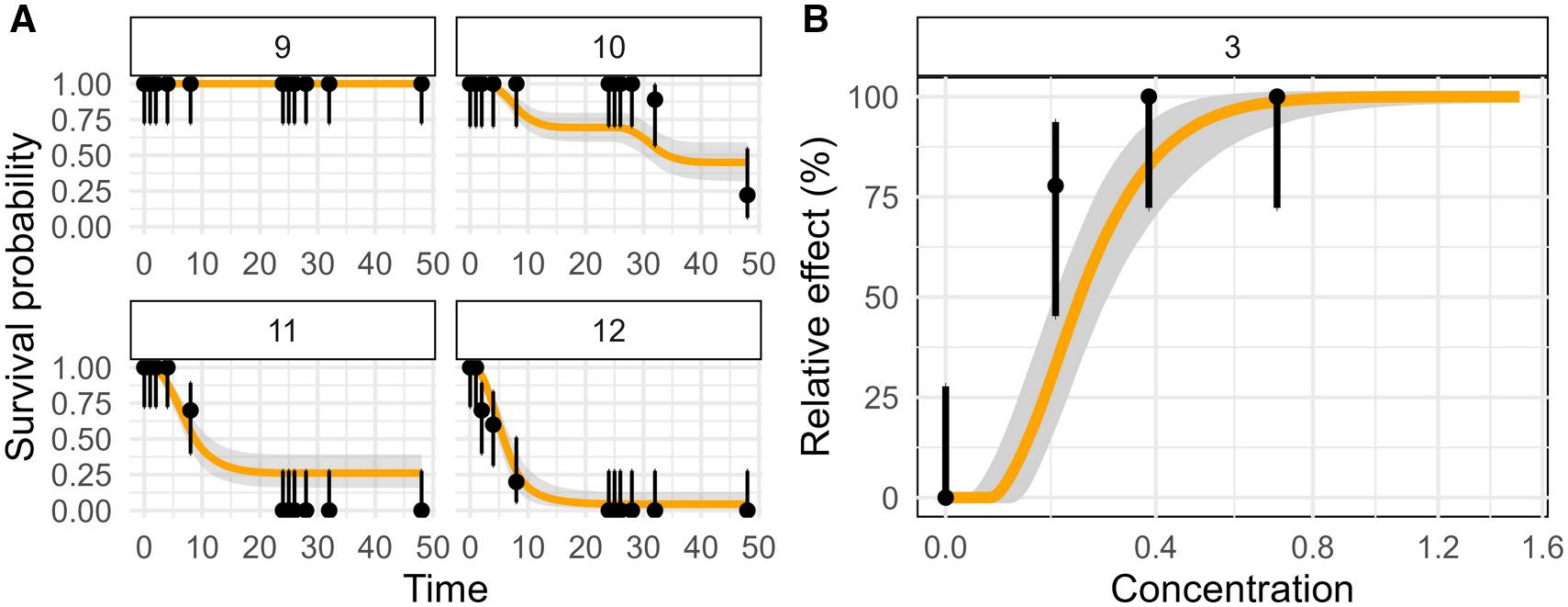
Example of model predictions presented in the online survey as (A) time-series (B) dose-response curve. Predictions and data used to create the graphs on (A) and (B) are identical (for B only data from the final time step is used). Further, in the survey, the image in (B) was of the same size as each panel in (A) to ensure comparable visibility of detail. Average goodness-of-fit based on normalised root-mean-square-error, posterior predictive check, survival probability prediction error (SPPE_min_), and SPPE_max_ was 0.86 (for the time series, panel A). Evaluators gave one score per image, i.e., for the four panels in (A) together only one score was given. Evaluator scores for the time series plots from six evaluations were 2, 2, 2, 3, 3, 4 and for the DRC plot from 5 evaluations were 2, 2, 3, 3, 4. Information provided to evaluators was (A) “This is a General Unified Threshold models of Survival (GUTS) model fit compared to validation data. Each panel shows survival probability over time for one treatment. Black dots and the corresponding error bars are observations with Wilson intervals. Orange lines are model predictions with credible intervals in grey around them. All model predictions are made by the same model. Concentration data is intentionally not shown.” (B) “These are dose-response curves derived from a GUTS model fit to validation data. Each panel corresponds to one experiment. Black dots are final survival probabilities plotted against maximum exposure concentrations for each treatment with error bars showing the Wilson intervals. Orange lines are model predictions with credible intervals in grey around them. All model predictions are made by the same model. As exposure profiles differed in duration and type of exposure among experiments, the predicted curves differ across panel(s).”

An additional aspect regarding visual assessments of model performance is that human perception of information is strongly influenced by how that information is presented (Cho et al., 2017; Dimara et al., 2019). General unified threshold model of survival model predictions are typically shown over time together with observed time series of survival (Figure 1A), whereas the statistical models underlying LD50 values are typically presented as dose-response curves (DRC; Figure 1B). Time series show more data and can visualize potential mismatches at intermediate time points. These temporal mismatches might be “compensated” by the end of the experiment and therefore not perceived in dose-response curves. Thus, such different representations of model results carry the risk of introducing cognitive bias when evaluating different modeling approaches.

The aim of our study was to test the extent to which quantitative GoF metrics agree with the visual assessments of evaluators on the temporal match of model predictions and data. To address this question, we conducted an anonymous online survey in which voluntary participants evaluated GUTS model predictions against observations from toxicity tests on a scale of 1 (best) to 6 (worst). We then compared the evaluations to GoF metrics calculated for the same model outputs and data. We assessed whether cut-off values could be derived for the quantitative GoF metrics at which evaluators agreed on fit acceptance. In addition, we assessed whether the same model fits were evaluated differently when presented as a dose-response curve compared with a time-series GUTS plot.

## Materials and methods

### GUTS model calibration and validation

General Unified Threshold models of Survival is a modeling framework to predict lethal effects from time-variable toxicant exposure. The reduced variant, GUTS-RED, obviates explicit consideration of internalized concentrations by modeling damage directly as a function of external exposure over time. This allows model calibration from standard toxicity tests that usually omit measurement of compound concentrations in the body. The probability of lethal effects is then modelled by one of two model variants: individual tolerance (IT) and stochastic death (SD). Individual tolerance assumes organism-specific tolerances to lethality of the compound. Stochastic death assumes a random mortality process, where organisms have the same probability to die at a given damage level. Further details on the well-established GUTS-RED are explained in Jager and Ashauer (2018a).

We calibrated and validated GUTS-RED models from 14 survival datasets. Each dataset comprised results from 3–7 experiments testing the effects of one specific compound on one species. The experiments considered a variety of exposure profiles, for example, acute or chronic as well as constant or pulsed exposure. All permutations of experiments for each of the data sets were used to create calibration and validation data. These data were used to construct both variants GUTS-RED-IT and GUTS-RED-SD, using the R-package *morse* (Baudrot & Charles, 2021). Data and model fitting procedures are described in detail in Bauer et al. (2024).

#### Quantitative GoF metrics

Several GoF metrics were calculated for all model predictions by Bauer et al. (2024). In our study, we restricted to those that Bauer et al. (2024) found most suitable for a parsimonious description of fit quality. Four of these GoF metrics were based on suggestions by EFSA PPR et al. (2018). These were NRMSE, PPC, SPPE-minimum across treatments and SPPE-maximum across treatments. Further, Nagelkerke pseudo-R^2^ was calculated for the end of experiments.

Normalised root-mean-square-error aggregates prediction errors over time and treatments, normalized by mean value of observations. Thus, it reflects prediction accuracy both for the level of mortality and its change over time.


(1) $$\mathrm{NRMSE}=100\%\cdot \frac{1}{\bar{Y}}\sqrt{\frac{1}{n}\sum_{i=1}^{n}{\left(y_{i}^{\mathrm{obs}}-y_{i}^{\mathrm{pred}}\right)}^{2}}$$

where *n* is the number of observations, $y_{i}^{\mathrm{obs}}$ is observed number of survivors at a given timepoint and treatment, $y_{i}^{\mathrm{pred}}$ is the predicted number of survivors at a given timepoint and treatment, and $\bar{Y}$ is the mean number of survivors across all observations.

Survival probability prediction error characterizes prediction accuracy at the end of the experiment. Two indices are calculated from SPPE, SPPE_min_ (describes the largest underestimation of effects) and SPPE_max_ (the largest overestimation of effects). Mathematically, ${\mathrm{SPPE}}_{\min}>0\%$ and ${\mathrm{SPPE}}_{\max}<0\%$ values are possible when survival probability is consistently overestimated or underestimated in all treatments. Such values are set to 0%, as in such cases there is no prediction error in either direction.


(2) $${\mathrm{SPPE}}_{k}=\frac{y_{k,\mathrm{tend}}^{\mathrm{obs}}-y_{k,\mathrm{tend}}^{\mathrm{pred}}}{y_{k,t0}}\cdot 100\%$$

(3) $${\mathrm{SPPE}}_{\min}=\underset{k}{\min}\left({\mathrm{SPPE}}_{k},0\%\right)$$

(4) $${\mathrm{SPPE}}_{\max}=\underset{k}{\max}\left({\mathrm{SPPE}}_{k},0\%\right)$$

Here, *k* is the treatment index, $y_{k,\mathrm{tend}}^{\mathrm{obs}}$ is the observed number of survivors at the last count of treatment *k*, $y_{k,\mathrm{tend}}^{\mathrm{pred}}$ is the predicted number of survivors at the last count of treatment *k*, and $y_{k,t0}$ is the number of tested organisms per treatment *k*.

Posterior predictive check is the percentage of observation data points the model can correctly predict within its 95% prediction credible interval.


(5) $$\mathrm{PPC}=\frac{100\%}{n}\sum_{i=1}^{n}I\left(y_{i}^{\mathrm{obs}}\in {CI}_{i}^{\mathrm{pred}}\left(95\%\right)\right)$$

where *n* is the number of observations, $y_{i}^{\mathrm{obs}}$ is the observed number of survivors at a given timepoint and treatment, ${\mathrm{CI}}_{i}^{\mathrm{pred}}$ (95%) is the 95% credible interval of predictions of the number of survivors at a given timepoint and treatment, and I(*xϵ[A; B]*) is an index function evaluating to 1 if *A ≤  x ≤B*, otherwise 0.

Nagelkerke pseudo-*R^2^* expresses the likelihood of the fitted GUTS model compared with the null model.


(6) $$R_{N}^{2}=\frac{1-{\left(\frac{L_{0}}{L_{M}}\right)}^{\frac{2}{n}}}{1-{\left(L_{0}\right)}^{\frac{2}{n}}}$$

where *n* is the number of treatments, *L_M_* is the likelihood of the calibrated model, and *L_0_* is likelihood of the null model (assuming the average survival probability calculated as the ratio of the sum of survivors and tested organisms over all treatments). The likelihood is calculated assuming a binomial distribution *L = ∑^n^B(k = $y_{i}^{\text{obs}}$; N = n*; *p*), where $y_{i}^{\mathrm{obs}}$ is the observed number of survivors in the last count of treatment *i* and *p* is the predicted survival probability for the last count.

### Selection of model predictions

From the 772 model calibrations and 6,852 model validations generated by Bauer et al. (2024), we selected model predictions for our study such that different levels of GoF, estimated for time-series, were approximately equally covered (See online supplementary information S1). Model predictions were subsequently filtered so that an effect of ≥ 70% was present in at least one of the treatments. We further excluded experiments with only one treatment. The filtering increases realism of the survey, as experimental data not showing effects or including only one treatment would not be used in practice to evaluate model performance.

### Visual evaluation via an online survey

We presented the selected model predictions in an anonymous online survey (See online supplementary information S2) to voluntary participants who visually rated the quality of the model fits to calibration or validation data. The images for visual rating showed model predictions versus the respective empirical data.

Two kinds of model fit images were presented for evaluation: a time-series figure and a dose response curve. The time-series figure showed the empirical survival over time (expressed as percentage of living individuals) against the temporally resolved model prediction as median curve and 95% credible band. Each exposure profile and treatment-level was presented separately in individual panels (Figure 1A). The DRC showed empirical and model-predicted survival at the end of the experiment and was plotted against treatment levels (Figure 1B). Dose-response curve from different experiments were displayed in separate panels, which allowed jointly displaying outcome of experiments with differing exposure profiles. For details on DRC predictions, see online supplementary information S3. Importantly, each calibrated GUTS model was used to predict time-series and DRCs such that both types of figures were based on the same information from toxicity experiments and only the statistical aggregation (visualization) differed. With this setting, we aimed at testing whether different forms of data aggregation affected qualitative assessment of model performance. In sum, all evaluators were shown 20 images in this order: five model calibrations as time series, five model calibrations as dose response curves, five model validations as time series, and five model validations as dose-response curves. In each category, images were drawn randomly and independently such that each evaluator was shown a different sample of images. All panels in the images were scaled to equal size to avoid bias on the visibility of details among the images.

**Figure 2.**
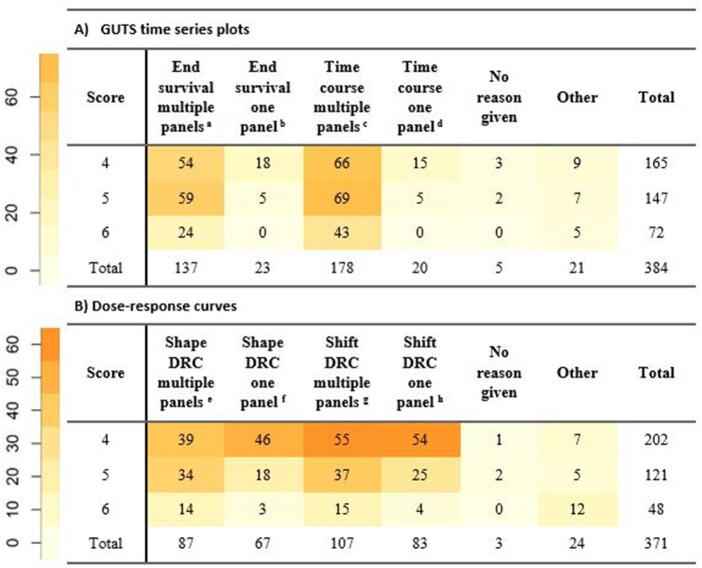
Number of cases where a given reason was selected as reason for nonacceptance of the model fit (i.e., score 4–6), presented as (A) time-series (B) dose-response curves. Exact text in survey: ^a^ Survival at the end of the test is not adequately captured (multiple panels). ^b^ Survival at the end of the test is not adequately captured (one panel). ^c^ Time course of the effect is not adequately captured (multiple panels). ^d^ Time course of the effect is not adequately captured (one panel). ^e^ Shape of dose-response curve not adequately captured (multiple panels). ^f^ Shape of dose-response curve not adequately captured (one panel). ^g^ Shape of dose-response curve captured but curve is shifted (multiple panels). ^h^ Shape of dose-response curve captured but curve is shifted (one panel). *Note*: Exact text in survey. GUTS – General Unified Threshold models of Survival; DRC = dose-response curve. To highlight patterns in the table, cells were colored. Higher numbers in the table correspond to darker colors (see color legends to the left).

In the survey, evaluators were given a brief introduction to the aims of the project and were asked to provide their affiliation category (Table 1) and their modeling experience (Table 2). Thereafter the images were presented together with the information whether they represented model calibration or validation. The participants could score the images on a scale of 1–6 (1: “excellent”, 2: “good”, 3: “adequate”, 4: “near acceptable”, 5: “poor”, 6: “no resemblance”). It was indicated to the evaluators that scores 1–3 are interpreted as acceptance and 4–6 as rejection of the model. If a score 4–6 was given, evaluators were given the option to record the reason for their nonacceptance of the model. They could choose between four previously formulated reasons (Figure 2 and online supplementary material Figure S2-1) or enter a custom reason.

**Table 1.** Sector affiliation of survey participants.

Sector category	Number of participants
Academia	15
Authority	5
Contract research organization (CRO)	13
Industry	24
Other	6
Not specified	1

**Table 2.** Experience of survey participants.

Model experience category	Number of participants
Modeler (experience in calibrating/validating TKTD models)	14
Modeler (without experience in calibrating/validating TKTD models)	16
Experienced model user (experience in evaluating reported model outputs)	12
Somewhat experienced model user (some familiarity with model outputs)	14
Nonmodeler (no or little experience with models)	8

TKTD = toxicokinetic-toxicodynamic.

It was deemed unnecessary and even counterproductive to test the participants’ answering behavior, for example, by repeatedly showing the same figures, because it could be assumed that practitioners were motivated to take part in a survey that could benefit their field. The participants could evaluate the figures at their own pace. Thus, participants with less experience in GUTS modeling had time to familiarize with the figures before rating fit quality. However, it cannot be excluded that participants “trained” during evaluation of the 20 figures, and later evaluations were influenced by figures that the participants had evaluated before.

The online survey was created with R *shiny* (Chang et al., 2022), deployed on shinyapps.io, and set up to anonymized store the survey data in a remote database. The link to the survey was circulated over several relevant mailing lists (e.g., SETAC Effect Modelling Interest Group, Pesticide Fate Modelling) and across the authors’ professional networks and social media (LinkedIn). The survey was open from December 21, 2022, to February 15, 2023.

### Statistical modeling

We conducted statistical modeling in two steps. In both steps, we fitted a Bayesian multinomial ordinal regression model with the evaluator score as response variable. “Score” was considered as an ordered categorical outcome variable, having a latent normal distribution across categories. Accordingly, we modelled Score with a cumulative model (Bürkner, 2021; Bürkner & Vuorre, 2019).

First, we determined which GoF metric or combination of GoF metrics best predicts the scores when using no other predictors. For this aim, we iteratively fitted 31 models using only one predictor. This predictor was either an individual GoF metric or a combined metric. Prior to combining metrics, they were rescaled to values between 0 and 1. Values of metrics where lower values mean better performance (NRMSE and SPPE) were inverted. Subsequently, we combined the metrics. We considered two approaches to combine metrics. In the standard scientific approach, we averaged the metrics. In the approach inspired by regulatory risk assessment, we conservatively considered the lowest value of the combined metrics (i.e., the worst assessment of the metrics). The combined GoFs were always a metric ranging between 0 and 1, with higher values indicating a model curve better fitting the predictions. We compared the resulting models using the ELPD-LOO values, which are the Bayesian leave-one-out cross-validation estimates of the expected log pointwise predictive density (Vehtari et al., 2017). Based on this step, we selected the best performing combination of metrics for each of the two approaches, called average GoF and minimum GoF, respectively. Subsequent analyses based on the combined metrics average GoF and minimum GoF were conducted separately for each of the two metrics.

Second, we constructed a more detailed model to predict evaluator scores based on the fixed effect predictors “average GoF” or “minimum GoF” (continuous), “image type” (calibration or validation, categorical), “evaluator affiliation” (categorical), “evaluator modeling experience” (ordered categorical) and the random effect “evaluatorID” (categorical). Thresholds to separate the ordered scores were allowed to vary between scores. The model assumed an interaction between the combined GoF metric and image type as well as between the combined GoF metric and experience. The first interaction reflects the hypothesis that calibrations and validations may be evaluated differently for poorly or well-fitting models. The second reflects the assumption that experienced modelers may differ in their evaluations from less experienced modelers or model users for poorly or well-fitting models. We applied the *brms* R package (Bürkner, 2017, 2018) implemented in Stan (Carpenter et al., 2017) for statistical modeling.

Third, we analyzed how the separate and combined metrics related to acceptance by evaluators (fits scored 1–3) or nonacceptance (fits scored 4–6). For this purpose, we modelled the binary acceptance response assuming a Bernoulli process using a logit link. Based on the effects of the GoF metrics contribution to explain evaluator acceptance, we predicted thresholds for each of the metrics. The GoF thresholds indicate at which values GoF metrics correspond to acceptance by 50% of evaluators.

Next, we investigated the reasons evaluators gave for nonacceptance of fits. To understand the importance of the various rejection reasons, we counted how often each reason was given to justify one of the rejection scores 4–6. In addition, we analyzed whether reasons for fit rejection correlated with fit quality metrics. We hypothesized that metrics evaluating the end of experiments (SPPE_min_ and SPPE_max_) would be correlated with the evaluators’ indication that the fits failed at the end of the time series (reasons a and b in section A of Figure 2). Similarly, we expected that metrics, which consider the whole time series (NRMSE and PPC), should correlate with reasons describing mismatch over the course of time (reasons c and d in section A of Figure 2). To conduct the analysis, we scaled the GoF metrics as described above, such that higher metric values indicated better fits. The reasons ticked by evaluators were grouped into “end of test-related” (reasons a and b) and “not related to the end of the test” (reasons c and d). The grouping was used as binary response variable and modelled with the four GoF metrics assuming a Bernoulli process using a logit link. Negative regression coefficients of this model indicate that the respective GoF metric in line with the evaluator perception judges a fit as bad because of mismatch at the end of the toxicity test. Positive regression coefficients indicate that the end of the toxicity test was not a sufficient criterion for the GoF metric to evaluate a fit as bad. Instead, the metric considered survival data over time.

Finally, we assessed how visual presentation (GUTS fits presented as a time series or as DRC) affected evaluator assessment. The analysis was conducted for each GUTS fit separately, to identify how different visual representations of the same fit were evaluated. For this purpose, in addition to the time series representation, we plotted the GUTS predictions for survival at the end of the experiments in the form of a DRC. General Unified Threshold models of Survival predictions instead of the empirical measurements were used to ensure that the DRC and time series representations were based on the same information content. We compared between DRC and time series presentations, whether a fit received a higher median score as well as whether more evaluators accepted a fit.

Fit acceptance for an image was calculated as the number of evaluations that accepted the image (scores ranging from 1 to 3) divided by the total number of evaluations of the image. This analysis was conducted visually, because the low number of evaluations per image impeded modeling the impact of timeseries versus DRC representation stratified by GUTS fits. Nevertheless, to underpin the per image conclusions quantitatively, we additionally modelled the probability of fit acceptance without separating by image. In the generalized linear model (evaluated with the *brms* R package), the binary acceptance response was transformed via a logit link and modelled dependent on the presentation as DRC or time series.

## Results and discussion

### Survey participants

A total of 74 model fits of calibration time series and 89 validation time series, 71 calibration DRCs, and 87 validation DRCs were assessed visually by 64 participants. Most images were evaluated by 3-4 participants. Participants came from all sectors, mainly from industry (37.5%), academia (23.4%) and contract research organizations (20.3%), and few from authorities (7.8%; Table 1). Experience levels were distributed relatively equally among those who filled out the survey (Table 2).

### GoF metrics in relation to visual scores

Using Bayesian LOO estimates, we compared prediction accuracies of 32 models predicting individual visual evaluation scores from a single GoF metric or combinations of GoF metrics (online supplementary material Table S4-1; for the relation of each of the single GoF metrics to the evaluator scores, see online supplementary material Figure S4-1). Best performing was an average of the scaled NRMSE, PPC, and SPPE_min_ values (ELPD-LOO: –892.15, SD: 15.5). The second highest and almost identical ELPD-LOO (ELPD-LOO: –893.27, SD: 15.5) was achieved when including SPPE_max_ in the average as well. Thus, considering SPPE_max_, with higher values indicating an increasing overprediction of effects, did not relevantly contribute to prediction accuracy. Including Nagelkerke-pseudo-*R^2^* in the average worsened predictions of scores compared with the previous models (ELPD-LOO: –915.53, SD: 15.16). Therefore, we excluded Nagelkerke-*R^2^* from further analysis, despite findings that Nagelkerke-*R^2^* can contribute to explain fit quality (Bauer et al., 2024). Hereafter, “average GoF” refers to the average of the scaled NRMSE, PPC, SPPE_min_, and SPPE_max_. Survival probability prediction error_max_ was included to retain a metric describing the overprediction of effects. When the GoF metrics were aggregated by their minimum, the combination of scaled NRMSE, PPC, SPPE_min_, and SPPE_max_ best predicted the evaluators’ scores (online supplementary material Table S4-2), confirming that these metrics are relevant to describe evaluator assessment.

Our results indicate the importance of the closeness of survival prediction and data (measured by NRMSE), the certainty of matching the survival data (PPC), and accurate predictions of survival at the end of an experiment (SPPE). These criteria capture relevant aspects of the form of survival time series from toxicity studies in ERA. Survival probability prediction error ensures consistency with standard ERA metrics that measure effects at the end of toxicity tests. Normalised root-mean-square-error accounts for prediction accuracy and PPC for uncertainty in the model prediction. However, PPC considers uncertainty only in calibration data, whereas uncertainty in validation data is ignored. Therefore, an accurate and precise prediction from a well-calibrated (i.e., calibrated to reliable data) GUTS might only slightly miss weaker validation data, which nevertheless results in low PPC.

Generally, the applicability of specific metrics for fit quality assessment depends on the purpose of the analysis, the type and quality of the data, and the shape of the fitted curve. For example, the same metrics have been suggested for prediction assessment of other TKTD models, but outcome of tests on performance and adequacy of the metrics for these models might alter the recommendation in the future (EFSA PPR et al., 2018).

### Other predictors of visual assessment scores

**Figure 3.**
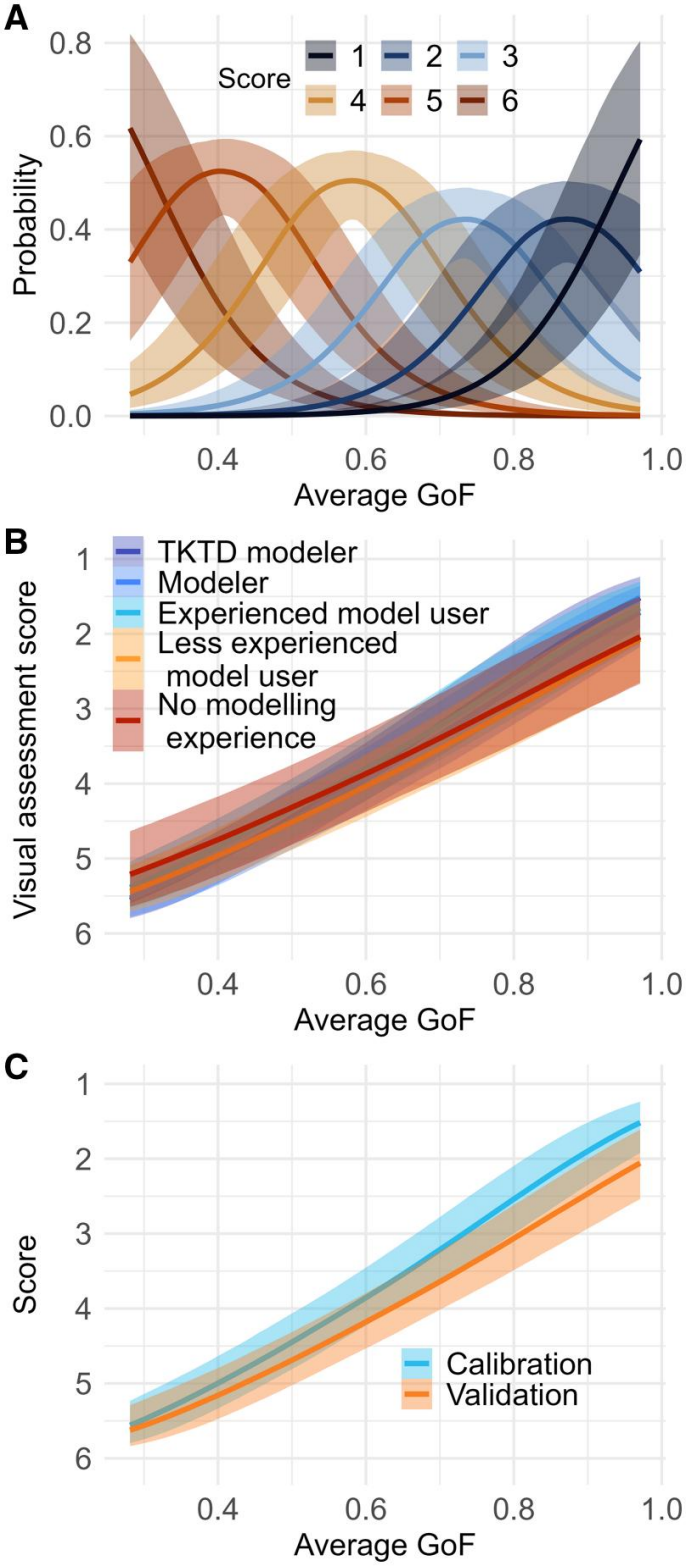
Predictions by the statistical model including all predictors. (A) Probability of an image receiving a given visual assessment score (red colors: scores indicating non-acceptance, 4–6; blue colors: scores indicating acceptance, 1–3) depending on average goodness-of-fit (GoF). Effects on predicted visual assessment scores of the interactions between average GoF and (B) modeling experience or (C) fits to calibration or validation data. For easier visualization, scores are handled as continuous instead of ordinal variables. The prediction lines and 95% CIs are generated from the regression curve for the indicated predictors, whereas other predictors are kept at their reference values (see online supplementary material Table S5-1). Random effects are excluded. For more information see the help page on function “conditional_effects” in the *brms* R package (Bürkner 2018). TKTD = toxicokinetic-toxicodynamic.

Goodness-of-fit of model predictions was strongly related to evaluators’ ratings (Figure 3A, online supplementary material Figure S4-1). The probabilities of lower scores (i.e., a better evaluation) increased with increasing GoF, showing a general agreement between visual perception and quantitative assessment of model quality. In the full statistical model (online supplementary material Table S5-1), the average GoF metric and the interaction between average GoF and modeling experience were significant (i.e., the 95% credible interval (CI) of the regression coefficients did not contain zero). The contribution of other factors was insignificant (the 95% CI contained zero).

For very high or very low GoF, agreement of evaluator scores was strongest (e.g., online supplementary information S6.1). Nevertheless, at intermediate average GoF, the scores were uncertain, reflecting some variability in perceptions of model performance. Particularly for average GoF in the range of 0.5–0.8, the probability distributions for different scores overlapped (Figure 3A). For example, at average GoF approximately 0.65, scores from 2–5 were chosen with at least a probability of 10%, and a score of 4 (“near acceptable”) had a 10% probability even for quantitatively highly ranked predictions with a combined GoF of around 0.8 (online supplementary information S6.2). The variability in scoring was found for the combined metrics average GoF (Figure 3A) and minimum GoF (online supplementary material Figure S5-1A), as well as the five single GoF metrics (online supplementary material Figure S4-1).

Toxicokinetic-toxicodynamic modeling experts and nonexperts perceived fits with higher average GoF values to be better (Figure 3B and online supplementary material Table S5-1). The similarity of judgements of experts and nonexperts indicates that the visual quality of GUTS model fits seemed intuitively clear without specialized training. However, evaluators with more modeling experience more likely assigned extreme scores (1 or 6) than less experienced evaluators (Figure 3B, online supplementary material Figures S5-2 and S5-3, and the significant interaction effect of modeling experience and average GoF, online supplementary material Table S5-1). Thus, modeling experience seemed to increase the self-trust in rating fits as excellent or completely failed. The minor difference in the evaluation of extreme cases was not significant when considering minimum GoF instead of average GoF (95% CI of model experience and its interaction with minimum GOF included 0; see online supplementary material Table S5-2), nor did it have an influence on model acceptance (online supplementary material Table S5-3).

There was an insignificant trend that calibration fits were scored better than validation fits when using average GoF as predictor (Figure 3C and online supplementary material Table S5-1). However, scores were significantly higher for validation plots (i.e., validation fits were considered worse overall) when minimum GoF, rather than the average, was used as the model predictor (online supplementary material Table S5-2). The significant interaction of minimum GoF and calibration/validation also suggests that calibration fits with low minimum GoF were rated worse than quantitatively equivalent fits for validation (online supplementary material Table S5-2 and online supplementary material Figure S5-1B and C). This suggests that the expectation that validations would be judged more leniently (because they were not calibrated to the data) was potentially correct when poor fits (in terms of minimum GoF) were considered. In general, evaluators likely applied the same quality criteria to calibration and validation fits.

The subjectivity of evaluators played a relevant role in scoring, as the random effect of evaluators was significant (online supplementary material Tables S5-1 and S5-2) and interpersonal variability of scores for the same image was relatively large (Figure 3; see online supplementary material S6.1 for an example). Such variability between evaluations of the same model in a risk assessment context would mean that the evaluator’s personal perception is a major contributing factor to acceptance of a model, introducing subjectivity into the process. One of the reasons for the large variation may be that evaluators in this survey were not provided with background information on how the models would be used. If evaluators assumed different model purposes as basis for their rating, they likely weighed different types of fit discrepancies differently. This was supported by the variety of reasons given by evaluators for rating fits unacceptable (see *Reasons for nonacceptance*). General Unified Threshold Model of Survival models can potentially be used for multiple purposes in the risk assessment, for example, used in themselves to derive toxicity endpoints under time-variable exposure scenarios (Gabsi et al., 2019) or as a building block in population models (Thursby et al., 2018). Intended purpose can have an influence on when the model is considered fit for that purpose (Jager & Ashauer, 2018b). Therefore, information about the planned uses of the model could have introduced more consistency across evaluator scores. Further, score variability might be slightly influenced by evaluators “learning” during the survey such that ratings might have been adjusted for later presented fits by comparing to previously experienced fits.

### Fit acceptance

**Figure 4.**
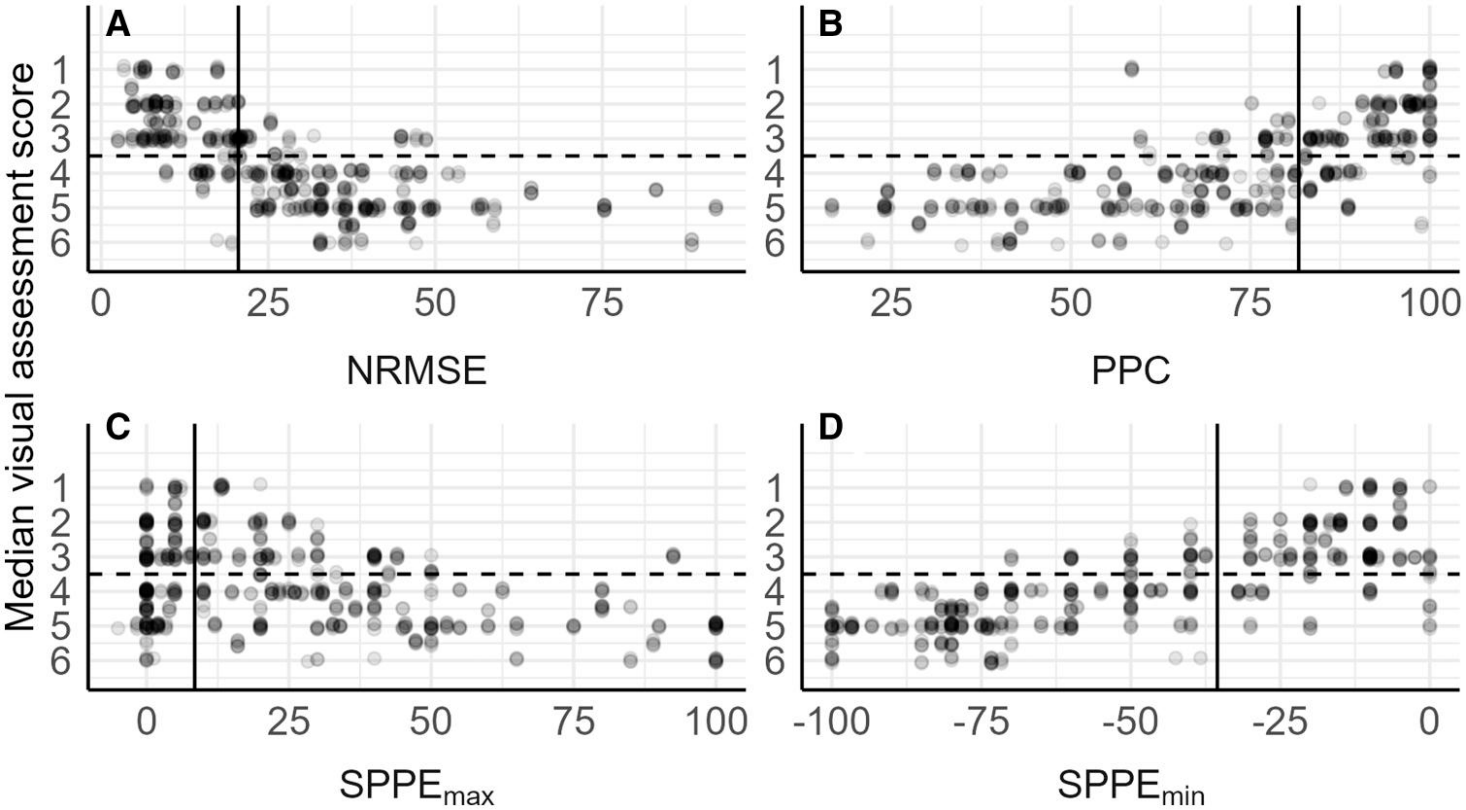
Visual assessment scores (median across evaluators for each image) and corresponding goodness-of-fit (GoF) metric values for the four GoF metrics that in combination are the best predictors of visual scores. Dot density is indicated by the grey scale (darker grey indicates higher dot density). Lower visual assessment scores indicate a qualitatively better fit perceived by the evaluator. The horizontal dashed line indicates the separation between scores indicating model acceptance (1–3) and nonacceptance (4–6). The vertical solid lines show the metric value at which 50% of evaluators were predicted to accept the fit (see online supplementary information S7). For normalised root-mean-square-error (NRMSE; A) and survival probability prediction error (SPPE_max_; C), lower values indicate better fit; for posterior predictive check (PPC; B) and SPPE_min_ (D) higher ones indicate better fit. A small vertical random jitter is applied on the points for better visibility.

Model predictions with GoF values indicating very good model performance of NRMSE (low means good), PPC (high means good), and SPPE_min_ (high means good) were usually rated acceptable (score 1–3, Figure 4). Model predictions with GoF values indicating weaknesses generally received a score of 4–6. Scoring was most variable for predictions with intermediate GoF values.

The easiest way to use metrics for decision-making reproducibly is to define cut-off values above or below which a model is considered acceptable or not. Thresholds for GoF metrics could be defined by a majority decision of evaluators, i.e., at least 50% of evaluators would accept the fits. The estimated thresholds for each of the single GoF metrics were NRMSE: 20.5%, PPC: 81.7%, SPPE_max_ = 8.45%, and SPPE_min_ = –35.5% (Figure 4, online supplementary material Table S7-1). Thus, single GoF metrics must be rather good (i.e., low for NRMSE and SPPE_max_, high for PPC and SPPE_min_) to reflect a visual fit-acceptance of the majority of evaluators. If the joint GoF metrics were considered as the criterion, we found that an average GoF above 74% or a minimum GoF above 54% of its range would be considered acceptable by 50% of the evaluators (online supplementary material Table S7-1). The thresholds for the joint metrics can be expected to be “lower” compared with the thresholds of single metrics, because fulfilling conditions on four metrics jointly is more demanding than fulfilling the single condition for each metric separately. Notably, the threshold for the minimum GoF coincides well with the regulatory suggestion that jointly NRMSE ≤ 50% and PPC ≥ 50% (EFSA PPR et al., 2018). As the minimum GoF further includes the SPPE metrics, our result suggests that these should not exceed the range of –50% to 50%.

### Reasons for nonacceptance

The main reason indicated for nonacceptance was “time course of the effect is not adequately captured (multiple panels)”, with “survival at the end of the test is not adequately captured (multiple panels)” as second (section A of Figure 2). There were fewer cases when the reason for nonacceptance was that time course or survival at the end were not captured in one panel or other reasons.

When issues were noted in multiple panels, an equal number of cases received scores 4 and 5, and approximately half as many cases a score of 6. On the other hand, when only one treatment was not well predicted, mostly a score of 4 was selected, that is, the model was evaluated more leniently. In summary, the more treatments and time points were missed by the model, the worse it was judged to have performed.

The rejection reasons for GUTS fits were correlated to specific GoF metrics (Table 3). There was a trend that SPPE metrics evaluated fits worse if they were rejected because of a mismatch at the end of the time series. Normalised root-mean-square-error and PPC, instead showed significant correlation to rejections because of mismatch along the time series. The findings demonstrate complementarity of metrics and justify their joint consideration in environmental risk assessment (EFSA PPR et al., 2018).

**Table 3.** Estimated impact of fit quality metrics on either rejecting a fit due to mismatch at the end (rejection reason a or b in Figure 2A) or throughout the time series (rejection reason c or d).

**Scaled metric** ^a^	Estimate	Estimated error	Lower 95% CI	Upper 95% CI
**Intercept**	−0.954	0.514	−1.975	0.034
**SPPE_min_**	−0.743	0.534	−1.821	0.311
**SPPE_max_**	−0.654	0.373	−1.392	0.084
**NRMSE**	1.397	0.724	0.039	2.858
**PPC**	1.028	0.519	0.038	2.045

*Note.* CI = credible interval; SPPE_min_ = survival probability prediction error, largest underestimation of effects; SPPE_max_ = survival probability prediction error, largest overestimation of effects; NRMSE = normalized root-mean-square-error; PPC = posterior predictive check.

a Metrics were scaled to a range of 0 to 1, where 0 indicates the worst evaluation of the fit and 1 the best. Thus, negatively estimated coefficients indicate that a metric considered a fit worse if it was rejected because of mismatch at the end of the fit. Positively estimated coefficients indicate that a metric considered a fit worse if it was rejected because of mismatch throughout the time series.

For DRCs (sec. B of Figure 2), the most common reason selected for nonacceptance was “shape of dose-response curve captured but curve is shifted (multiple panels)”, followed by “shape of dose-response curve not adequately captured (multiple panels)” and “shape of dose-response curve captured but curve is shifted (one panel)”, then “shape of dose-response curve not adequately captured (one panel)”. Regarding the distribution of evaluations among individual scores, similar to the time-series evaluations, worse scores were given when multiple panels were not well predicted compared with only one panel.

The most common custom reasons named by evaluators were similar for calibrations and validations, and time-series and dose-response curves. In seven cases, the reason was “too few observation points,” or “too few observation points with high effects.” This indicated that the acceptance of a model was perceived to be inseparable from judging the quality of the underlying data. In fact, survival data covering a wide range of lethal effects has been shown to improve calibration of GUTS models (Bauer et al., 2024). Lack of information in data is evaluated by pseudo-*R^2^* metrics, such as the Nagelkerke pseudo-*R^2^*, which compare GUTS predictions considering differences among experimental treatments with predictions from the null-model that assumes an average survival probability across all treatments. If outcome of a toxicity test is uninformative (e.g., because the effect range is not well represented), the additional structural complexity of a GUTS model (relative to the null model) cannot be accurately calibrated. Hence, the GUTS model prediction would be no better than the prediction of the simpler null model. Although the lack of information was recognized as problematic by some evaluators in their custom comments, the pseudo-*R^2^* metrics did not relevantly contribute to explaining evaluator scores. We assume that only extreme lack of information content in calibration data is obvious to evaluators in visual inspections of fit graphs, which triggered the comments, and subtle prediction improvements of GUTS compared with the null model would not be perceived. It must be remarked that a lack of information content in toxicity data, additionally to TKTD modeling, affects other risk assessment statistics, too. However, the visual inspection of TKTD timeseries plots might increase the chance to identify the issue.

The second most common custom reason was a “too wide credible interval” (four cases). Wide credible intervals can be an issue for model acceptance, as they reflect an uncertainty in the calibration. Calibration uncertainty might arise from technical issues as well as insufficient or contradictory information in the calibration data set and therefore should be thoroughly investigated (Bauer et al., 2024). The possibility to highlight uncertainty in calibration or prediction is an advantage for assessing the reliability of risks and is a subject of active research (Ashauer et al., 2016; Klein, 2018). Several GUTS software packages already implement routines to include uncertainties in model predictions (Baudrot et al., 2018; Jager, 2021).

A third reason for rejection, mentioned by three evaluators, was that time course and survival at the end of the test were not captured and these equally contributed to their nonacceptance of the model. Other evaluators indicated that effects were consistently over- or underestimated. Such mismatches suggest that a model cannot appropriately describe the data, and prediction accuracy should be doubted. However, in a risk assessment context, if a model systematically overestimates mortality compared with the measured data, the higher sensitivity predicted by the model might be considered conservative.

### DRC and time-series representations

**Figure 5.**
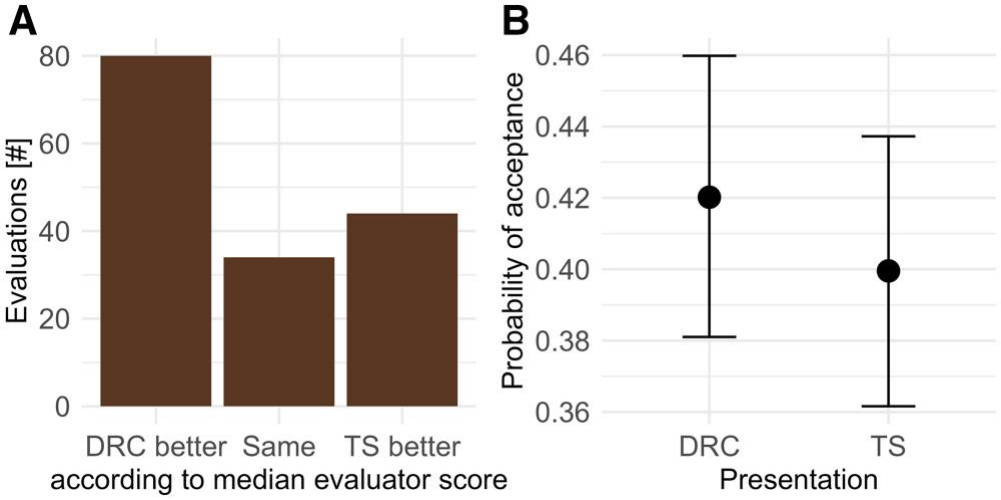
Comparison of the perception of dose-response curve (DRC) and time-series (TS) presentation of model predictions. (A) Number of evaluations where the DRC presentation of the same model predictions received better (i.e., lower), the same or worse (i.e., higher) median visual assessment scores than the TS presentation. (B) Predicted probability of model acceptance (score 1–3) by evaluators if fits were presented as DRC or TS.

In approximately 50% of the cases, model predictions displayed as dose-response curve were better rated than the corresponding time-series representation. In 22% of the cases, dose-response curve and time-series representation were equally rated, and in the remaining 28% of the cases, the time-series representation was better rated (Figure 5A). Similarly, there was a nonsignificant tendency that evaluators were more likely to accept fits displayed as DRC than as time series (Figure 5B). This indicates some bias in the perception of model results depending on the type of visual representation. Analyzing the fits separately (see online supplementary material Figure S8-1), ratings of DRC and time series coincided for about one third of the images. A different decision was found for only four images. In three out of the four cases, the DRC representation was accepted whereas the time series was not. However, the analysis is impaired by the low number of evaluations per image. To be conclusive, more evaluations would be needed, particularly for fits with intermediate fit quality.

In principle, time-series representations are more suited to represent GUTS model predictions than dose-response curves, as also noted by one evaluator, because they convey the temporal change in survival (Jager & Ashauer, 2018a). The temporal dynamics are hidden in DRCs that are constructed from survival at the end of an experiment only. This shortcoming of the DRC statistics arises even in the present study, where we took precautions for a fair comparison. Particularly, we constructed time series and DRC from GUTS predictions, ensuring in this way that the full information content of the empirical toxicity tests was considered by both methods. Standard practice of constructing DRC omits the GUTS modeling step and instead fits DRC directly to the empirically estimated survival at the end of the toxicity tests, thereby ignoring intermediary data points that would convey the temporal survival information.

Time series plots more transparently allow judgement of fit quality, because evaluators get better insight in potential mismatches between model predictions and data. We assume that the higher chance to spot discrepancies in time series plots compared to DRC ultimately led to a better rating of DRC compared with the time series representation.

## Conclusion

To summarize, we found for GUTS that the metrics suggested in EFSA PPR et al. (2018) reasonably correspond to visual assessment scores when used in combination, and the suggested thresholds in EFSA PPR et al. (2018) reflect a conservative evaluation. Evaluator affiliation had no significant influence, which fosters trust in an impartial model evaluation for risk assessment. Experience only had a small influence on scoring. However, there is an interpersonal variability not accounted for by affiliation or experience, potentially reflecting different individual expectations about how closely model predictions should fit the data. Individual expectations may have resulted from different assumptions about how the models would be used in risk assessment because the ERA purpose was not specified in the survey.

Further, the presentation of model predictions and data affected visual evaluation. The same model fits tended to be evaluated better when presented as a DRC plot than when presented as a time series GUTS plot. Naturally, the more complete information presented in time series GUTS plots allows evaluators to spot mismatches that are otherwise hidden, because DRC plots only show data from the end of an experiment. In this sense, time series GUTS plots better communicate uncertainty compared to visualizations as DRCs and therefore can provide more informed risk assessments. Additionally, to the better transparency of time series GUTS plots, current risk assessment schemes request similar or even higher proof of reliability for TKTD models (e.g., validation) compared with standard DRC models (EFSA PPR et al., 2018; EFSA Scientific Committee et al., 2022; Organisation for Economic Co-operation and Development [OECD], 2006). In accordance with OECD (2006), we suggest that, at least to analyze effects from time-variable exposure, TKTD approaches should be applied.

Our study underpins the process suggested in EFSA PPR et al. (2018) for the evaluation of TKTD models. Particularly, it reveals that the suggested complementary evaluation of three aspects is desirable: input data quality, visual representation of model fits to calibration and validation, and GoF metrics on calibration and validation. First, EFSA PPR et al. (2018) demands strict evaluation of input data characteristics to ensure sufficient information content for calibration and validation of TKTD models. These criteria, particularly the requirement for a wide effect range in toxicity tests, were also found important by Bauer et al. (2024). In this survey, necessity for adequate information content in toxicity data was confirmed by survey participants for visual fit assessment as well. Second, the visual representation of model fits provides insight in the temporal course of model performance and can reveal fitting mismatch to which quantitative GoF metrics might be “blind” because of their tailoring to specific fitting problems. However, the visual inspection is a subjective assessment, although assessments in the survey were impartial concerning the professional background of evaluators. Therefore, third, quantitative GoF metrics add a consistent baseline for model quality judgement that is independent of interevaluator differences. The metrics and cut-off criteria suggested for application in the European ERA of plant protection products might be adapted according to further experience with their application (EFSA PPR et al., 2018). In this context, our study shows that the metrics reflect evaluators’ decisions. A combination of the suggested metrics (NRMSE, PPC, and SPPE) best describes the rating of evaluators. Hence, the specific aspects of fit quality that the GoF metrics address seem relevant to evaluators, too.

Finally, EFSA PPR et al. (2018) suggested the quantitative cut-off criterion that NRMSE ≤ 50% and PPC ≥ 50% should both be fulfilled. We found that 50% of evaluators visually judged a GUTS time series fit acceptable if the minimum GoF exceeded 54%. This threshold, for the slightly more conservative metric minimum GoF that additionally includes the SPPE metric, closely matches the criterion suggested by EFSA PPR et al. (2018). Hence, our results give evidence that the criteria suggested by EFSA PPR et al. (2018) appropriately ensure reliable assessments of GUTS models.

Based on a survey among stakeholders from all institutions involved in ERA of PPP, it can be deduced that evaluation of input data quality, visual representation of model fit, and GoF metrics all play their role in providing a transparent picture of model quality. A model fulfilling the criteria laid out by EFSA PPR et al. (2018) should be trusted to provide robust reliable predictions for the application in the assessment of environmental risks from chemicals.

## Supplementary Material

vgae015_Supplementary_Data

## Data Availability

Anonymized extracts of the survey results, as well as scripts to reproduce figures and analyses presented in the manuscript are available from the corresponding author upon request (alexander.singer@rifcon.de).
