# Supplementary material for: Comparison of visual assessment and quantitative goodness-of-fit metrics on GUTS model fits
Source: Environ Toxicol Chem. 2025 Jan 6;44(1):240–50. doi: 10.1093/etojnl/vgae015 (PMC11790204; doi:10.1093/etojnl/vgae015)
Supplement: vgae015_Supplementary_Data [file vgae015_supplementary_data.pdf]

Supporting Information

for article:
Bauer B, Singer A, Jakoby O, Nickisch D, Preuss T, Witt J, Wittwer T, Gergs A. Comparison of visual assessment and quantitative goodness-of-fit metrics on GUTS model fits. Environ Toxicol Chem.
doi:10.1093/etojnl/vgae015.

S1. Average GoF distribution across images

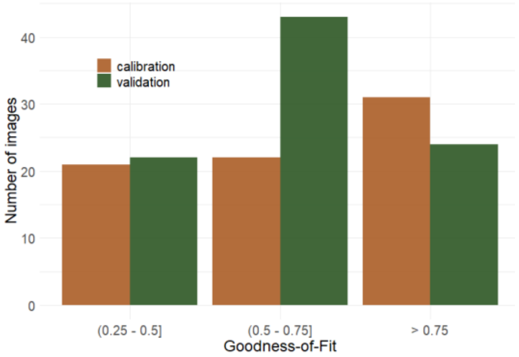

Figure S 1-1 Number of calibration (brown) and validation (green) time-series images with different Goodness-of-Fit levels evaluated in the survey. Validations of intermediate GoF are overrepresented.

S2. Survey structure

Survey introduction text

"First of all, thank you very much for participating in our survey. You'll be shown a series of five model fits to experimental data provided by Bayer for which you will judge whether the quality of each model fit is acceptable or not. The graphs contain GUTS model calibration and validation fits to multiple treatments from individual experiments as well as combinations of experiments. Graphs will be presented first in the form of time-series and second as dose-response curves.

All model analyses were conducted using the R-package morse (Baudrot & Charles, 2021)."

Professional background of survey participants

Participants were asked to provide their affiliation category with options:

- Academia
- Authority
- Contract Research Organisation (CRO)
- Industry
- Other
- Not specified

Further, participants were asked to provide their modelling experience with options:

- modeller (experience in calibrating/validating TKTD models)
- modeller (without experience in calibrating/validating TKTD models)
- experienced model user (experience in evaluating reported model outputs)
- somewhat experienced model user (some familiarity with model outputs)
- non-modeller (no or little experience with models)

Tasks to rate visual fit representations

Participants were asked to rate 20 images in this order: 5 model calibrations as time series, 5 model calibrations as dose response curves, 5 model validations as time-series, and 5 model validations as dose-response curves. For each image, participants were asked to give a score between 1 (excellent) and 6 (no resemblance). Only, if the score was ≥ 4 (fit considered not acceptable), the participants were additionally asked to give a reason for their decision from a set of predefined options. Alternatively, a customized reason could be typed in a free-text field. For detail, see Figure S 2-1.

Tasks:			
**Task 1: Rating of model fit, represented as time-series or dose-response curve**			**Task 2: Provision of reason, why a fit was rated "Not Acceptable"***
**Options to select from:**			
**Scores**	**Meaning**	**Risk assessment interpretation**	**Choices for reasons**
1<br>2<br>3	Excellent<br>Good<br>Adequate	Acceptable (Scores: 1-3)	
4<br>5<br>6	Near Acceptable<br>Poor<br>No resemblance	Not acceptable (Scores 4 – 6)	*Time-series*<br>- no reason given<br>- survival at the end of the test is not adequately captured (one panel)<br>- time course of the effect is not adequately captured (one panel)<br>- survival at the end of the test is not adequately captured (multiple panels)<br>- time course of the effect is not adequately captured (multiple panels)<br>- other: A free text field is available to specify the reason<br><br>*Dose response curve*<br>- no reason given<br>- shape of dose-response curve not adequately captured (one panel)<br>- shape of dose-response curve captured but curve is shifted (one panel)<br>- shape of dose-response curve not adequately captured (multiple panels)<br>- shape of dose-response curve captured but curve is shifted (multiple panels)<br>- other: A free text field is available to specify the reason

* If fits were rated "Acceptable", survey participants were not asked to provide a reason

Figure S 2-1 Order of tasks to be conducted for each displayed model fit, and response options.

S3. DRC construction

We constructed GUTS-predicted dose response curves for the toxicity experiments in several steps.

1.) We calculated an average exposure profile for the toxicity experiment by taking the mean concentration over all treatments for each concentration measurement. This was possible, because concentrations tended to be measured at similar times in the experiments.
2.) We multiplied the average exposure profile with a multiplication factor (Ashauer et al. 2013). We chose multiplication factors such that the concentration range of the experiment was covered. If 100% mortality was not reached at the highest concentration level, we increased multiplication factors until it was reached so a complete drc could be constructed. To be able to construct a smooth DRC, several intermediate multiplication factors were used.
3.) We used the calibrated GUTS-RED to predict survival at the end of each of the multiplied average profiles. This created survival predictions that corresponded to measured survival at the of the experiment.
   We applied R-function: morse:::MFx.survFit for model predictions.
4.) We plotted the predicted survival (median and 95%-CI) against the time-weighted average concentration of the profiles (maximum concentration for spiked-exposure studies). Similarly, we added the experimentally measured survival at the end of the experiment. Choice of the time-weighted-average procedure was pragmatic, in order to normalize the concentration scale of temporally variable exposure. This allowed matching the empirical and predicted survival as both were plotted against the similarly scaled concentration axis.

S4. Statistical models using GoF metrics as predictors

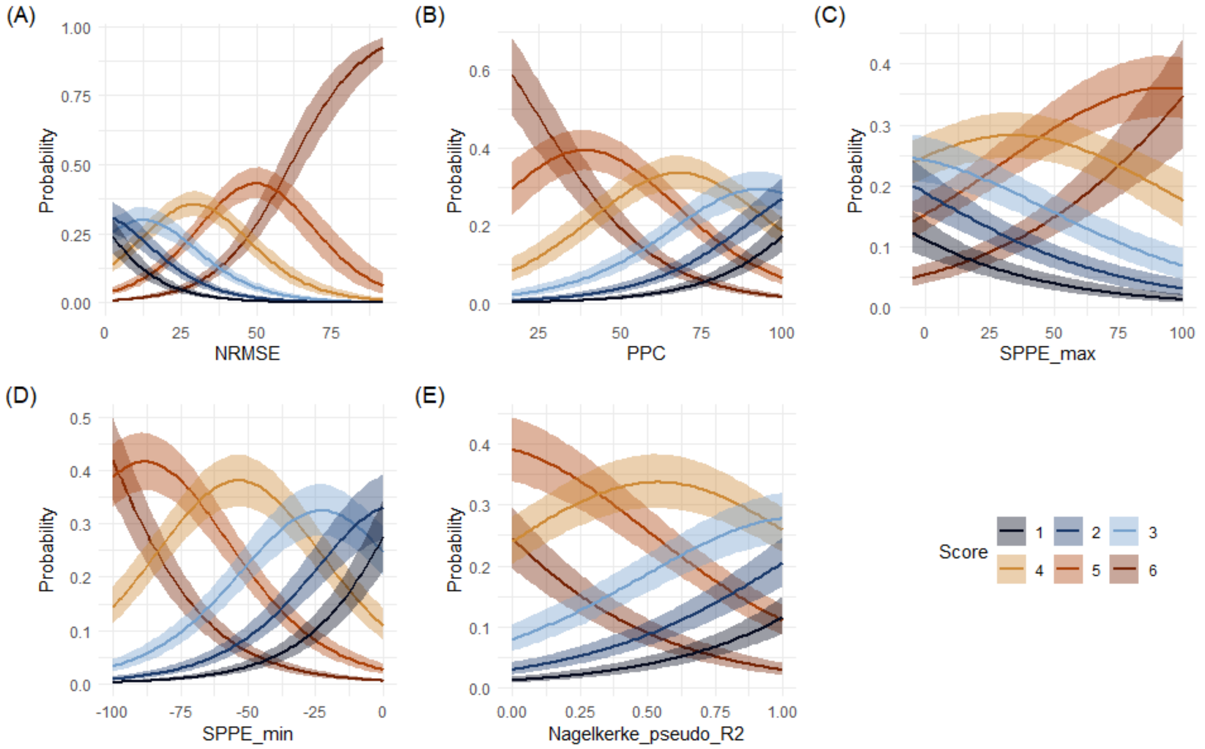

Figure S 4-1 Predictions by the statistical models including only one metric as predictor. Probability of an image receiving a given visual assessment score (red colors: scores indicating non-acceptance, 4-6; blue colors: scores indicating acceptance, 1-3) depending on A) NRMSE, B) PPC, C) $SPPE_{max}$, D) $SPPE_{min}$, E) Nagelkerke-pseudo-R^2. The prediction lines and 95% CI are predicted from the regression line at each value of the given metric.

Table S 4-1 ELPD-LOO estimates (the higher the better) and their standard deviations for all models using only a single or combined GoF metric as predictor (last column).

Estimate	SE	Metrics used to construct average GoF
-892.1465	15.49376	NRMSE_PPC_SPPE_min
-893.2691	15.48859	NRMSE_PPC_SPPE_max_SPPE_min
-904.0132	16.03163	NRMSE_SPPE_max_SPPE_min
-908.3705	14.66029	NRMSE_PPC
-910.0352	16.35188	NRMSE_SPPE_min
-915.5325	15.16019	Nagelkerke_pseudo_R2_NRMSE_PPC_SPPE_max_SPPE_min
-919.1646	14.91748	Nagelkerke_pseudo_R2_NRMSE_PPC_SPPE_min
-923.6417	14.78793	PPC_SPPE_max_SPPE_min
-924.1885	14.59731	PPC_SPPE_min
-925.9198	15.62228	Nagelkerke_pseudo_R2_NRMSE_SPPE_max_SPPE_min
-927.7217	14.58490	Nagelkerke_pseudo_R2_PPC_SPPE_max_SPPE_min

Estimate	SE	Metrics used to construct average GoF
-931.5277	14.30533	Nagelkerke_pseudo_R2_PPC_SPPE_min
-932.3721	15.39569	Nagelkerke_pseudo_R2_NRMSE_SPPE_min
-932.5977	15.07847	Nagelkerke_pseudo_R2_NRMSE_PPC_SPPE_max
-933.7738	14.85798	Nagelkerke_pseudo_R2_NRMSE_PPC
-937.4444	14.77195	SPPE_max_SPPE_min
-938.6870	15.75455	SPPE_min
-938.8797	14.62096	NRMSE_PPC_SPPE_max
-939.3296	14.99787	Nagelkerke_pseudo_R2_SPPE_max_SPPE_min
-945.9910	14.70364	Nagelkerke_pseudo_R2_SPPE_min
-948.5259	15.65021	Nagelkerke_pseudo_R2_NRMSE_SPPE_max
-949.6650	14.44287	Nagelkerke_pseudo_R2_PPC_SPPE_max
-951.1398	14.14233	Nagelkerke_pseudo_R2_PPC
-952.8829	15.48676	Nagelkerke_pseudo_R2_NRMSE
-957.5463	16.26547	NRMSE
-967.7716	14.88670	Nagelkerke_pseudo_R2_SPPE_max
-972.9218	14.70135	Nagelkerke_pseudo_R2
-985.2406	13.65141	PPC
-985.6033	14.45628	NRMSE_SPPE_max
-996.3011	13.76701	PPC_SPPE_max
-1063.2707	11.67525	SPPE_max

Replication with Minimum GoF

To test sensitivity of our analysis to the aggregation of the single GoF metrics, we used apart from the Average GoF a second metric: the Minimum GoF. We constructed the Minimum GoF as the minimum of NRMSE, PPC, $SPPE_{min}$ and $SPPE_{max}$, which best fitted evaluator ratings. Nagelkerke R^2 was omitted according to model selection (Table S 4-2).

Average and Minimum GoF provide slightly different insight on model fit performance. Average GoF considers the separate GoFs equally weighted. In contrast, Minimum GoF weights only the worst of the single GoF metrics, which can be seen as a worst-case approach that might be suited for risk assessment. Minimum and Average GoF particularly differ in the range of lower values (Figure S 4-2).

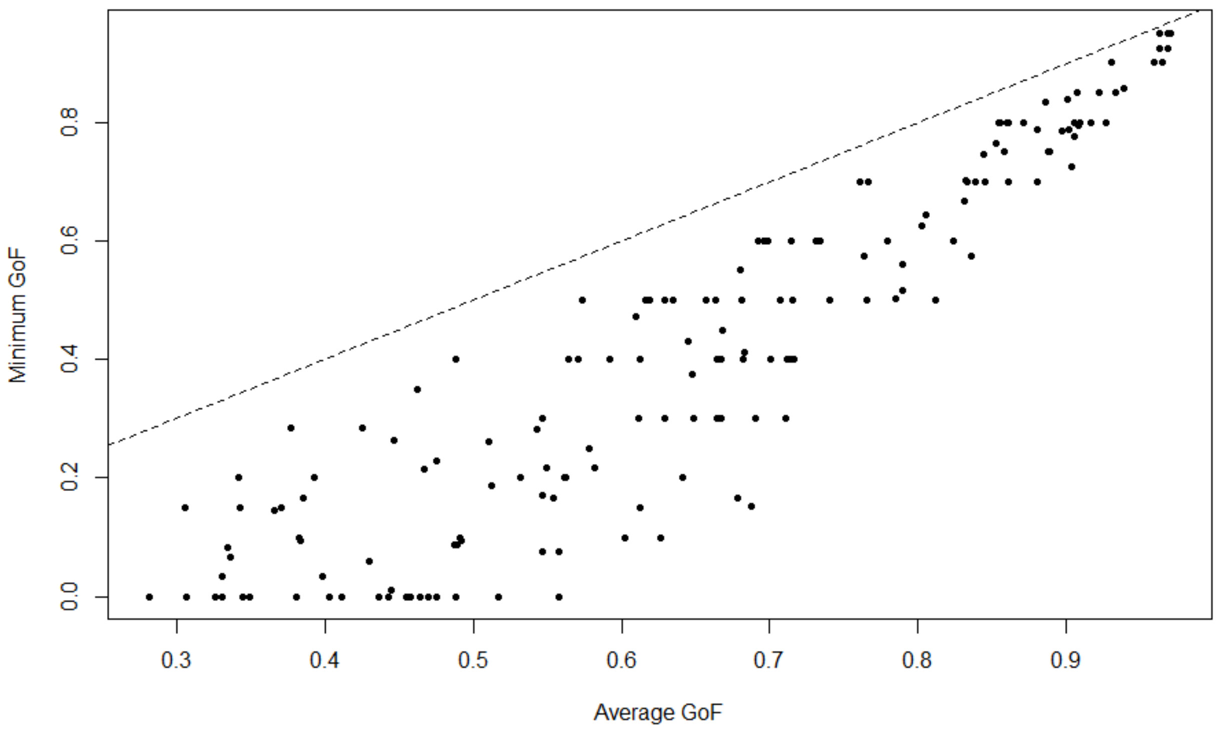

Figure S 4-2 Minimum versus average GOF. The 1:1 line is dashed.

Table S 4-2 ELPD-LOO estimates (the higher the better) and their standard deviations for all models using only a single or the Minimum GoF metric as predictor (last column).

Estimate	SE	Metrics used to construct Minimum GoF
-894.3884	16.16028	NRMSE_PPC_SPPE_max_SPPE_min
-895.1754	16.45505	NRMSE_SPPE_max_SPPE_min
-897.0057	16.51258	SPPE_max_SPPE_min
-897.3556	16.29176	PPC_SPPE_max_SPPE_min
-911.7944	15.66054	NRMSE_SPPE_min
-912.7450	15.32831	NRMSE_PPC_SPPE_min
-916.0731	15.77750	Nagelkerke_pseudo_R2_NRMSE_PPC_SPPE_max_SPPE_min
-918.0229	15.89128	Nagelkerke_pseudo_R2_NRMSE_SPPE_max_SPPE_min
-919.2243	15.23185	PPC_SPPE_min
-919.4764	15.73066	Nagelkerke_pseudo_R2_PPC_SPPE_max_SPPE_min
-920.9867	15.81223	Nagelkerke_pseudo_R2_SPPE_max_SPPE_min
-928.7452	15.34291	Nagelkerke_pseudo_R2_NRMSE_PPC_SPPE_min
-928.9362	15.21885	Nagelkerke_pseudo_R2_NRMSE_PPC_SPPE_max
-930.8320	15.49165	Nagelkerke_pseudo_R2_NRMSE_SPPE_min
-932.6179	15.24915	Nagelkerke_pseudo_R2_PPC_SPPE_min

Estimate	SE	Metrics used to construct Minimum GoF
-935.3733	15.06945	Nagelkerke_pseudo_R2_PPC_SPPE_max
-937.8079	14.92370	Nagelkerke_pseudo_R2_NRMSE_PPC
-938.6273	15.70247	SPPE_min
-939.4061	15.22214	Nagelkerke_pseudo_R2_SPPE_min
-941.7038	14.80046	Nagelkerke_pseudo_R2_PPC
-942.9185	14.49529	NRMSE_PPC
-944.5766	15.42087	Nagelkerke_pseudo_R2_NRMSE_SPPE_max
-945.0870	14.86613	NRMSE_PPC_SPPE_max
-955.9641	15.02814	Nagelkerke_pseudo_R2_NRMSE
-957.2520	15.14358	Nagelkerke_pseudo_R2_SPPE_max
-957.4193	16.25994	NRMSE
-971.9984	15.26265	NRMSE_SPPE_max
-974.1521	14.51842	Nagelkerke_pseudo_R2
-985.2211	13.65977	PPC
-990.5440	14.14451	PPC_SPPE_max
-1063.0657	11.66897	SPPE_max

S5. Full statistical model

We fitted a Bayesian multinomial ordinal regression model (estimated using MCMC sampling with 4 chains of 2000 iterations and a warmup of 1000) to predict visual assessment scores with average GoF (calculated from NRMSE, PPC, $SPPE_{min}$ and $SPPE_{max}$), image type ("img_type", calibration or validation), affiliation ("sector"), modelling experience (modExperience) and a random evaluator ID effect (formula: score ~ average_GoF + img_type + sector + mo(modExperience) + (1|evaluator) + average_GoF:img_type + average_GoF:mo(modExperience). Priors over parameters were set as default in the brms package. These are improper flat priors over the reals for the slopes and Student-t with 3 degrees of freedom for the intercepts and the standard deviations for the random effect (intercept and group-level effects). Finally, the simplex monotonic parameters, which are a special parameter vector to estimate the 'normalized distances' between consecutive predictor categories for the predictor modelling experience have a dirichlet prior. For more information on priors please see the function description of set_prior in the documentation of the brms package (https://cran.r-project.org/web/packages/brms/index.html).

Table S 5-1 Modelling evaluator scores with average GoF as one predictor: Estimates with 95% confidence intervals of model parameters, below model performance indicators.

	Estimate	2.5 %	97.5 %
Intercept[1][a]	4.29	3.00	5.66
Intercept[2][a]	6.72	5.40	8.11
Intercept[3][a]	9.02	7.61	10.53
Intercept[4][a]	10.90	9.39	12.51
Intercept[5][a]	12.8	11.2	14.5
average_GoF	13.6	11.8	15.6
img_typevalidation[b]	0.239	-0.877	1.374
sectorAuthority[c]	-0.158	-1.664	1.304
sectorCRO[c]	0.173	-0.906	1.206
sectorIndustry[c]	-0.203	-1.107	0.764
sectorNotspecified[c]	-1.08	-4.15	1.75
sectorOther[c]	-0.904	-2.203	0.504
average_GoF × img_typevalidation	-1.6216	-3.2745	0.0453
Experience (momod)[d]	0.49198	-0.00618	1.03841
Experience(momod) × average_GoF	-0.840	-1.488	-0.258
sd_evaluator__Intercept	1.239	0.964	1.619
simo_momodExperience1[1] [de]	0.14493	0.00592	0.54991
simo_momodExperience1[2] [de]	0.2728	0.0191	0.7050

	Estimate	2.5 %	97.5 %
simo_momodExperience1[3] [de]	0.08073	0.00308	0.43913
simo_momodExperience1[4] [de]	0.4140	0.0296	0.8003
simo_momodExperience × average_GoF1[1]	0.2556	0.0195	0.5776
simo_momodExperience × average_GoF1[2]	0.10655	0.00423	0.44158
simo_momodExperience × average_GoF1[3]	0.3533	0.0412	0.7195
simo_momodExperience × average_GoF1[4]	0.2165	0.0128	0.5773
Num.Obs[f].	640		
Expected log pointwise predictive density for a new dataset[g] (ELPD)	-800.5		
Standard error (ELPD s.e.)	17.8		
Leave-one-out cross-validation[g] information criterion (LOOIC)	1601.0		
Standard error (LOOIC s.e.)	35.7		

[a]Intercept: Thresholds that separate scores across the normally distributed latent variable (Bürkner and Vuorre 2019)

[b]img_type: presented to evaluators were either images of fits to calibration or to validation data

[c]sector: Sectors comprised academia, authority, contract research organistation (CRO), industry, other or not specified.

[d]Experience: Experience comprised modeller (experience in calibrating/validating TKTD models), modeller (without experience in calibrating/validating TKTD models), experienced model user (experience in evaluating reported model outputs), somewhat experienced model user (some familiarity with model outputs), non-modeller (no or little experience with models). The ordered factor experience was modelled as a monotonic predictor (Bürkner and Charpentier 2020).

[e]simo_momod: Simplex index for the monotonic predictor, which indicates the normalized distance to the next lower category (Bürkner and Charpentier 2020).

[f]Number of observations

[g]see (Vehtari et al. 2017)

Replication with Minimum GoF

When adding further factors (img_type: calibration/validation; sector: Academia, Authority, CRO, Industry, NotSpecified, Other; Experience: 1 (highest) – 5 (lowest), Minimum GoF and image type (calibration/validation) as well as their interaction showed significant effects (Table S 5-2 and Figure S 5-1). Fits to validation data were rated slightly worse compared to fits to calibration data (Figure S 5-1B). The interaction of Image type with Minimum GoF indicated a sharper evaluation of fits to calibration data, with a tendency to consider extreme scores for obviously good or bad fit, i.e. fits

with both high/low score and high/low Minimum GoF (Figure S 5-1C). Also significant was the random effect of evaluators indicating variance in subjective individual evaluations. Visual inspection of the influence of sector and modelling experience indicated statistically insignificant trends. Increasing group-sizes for these multi-level factors, i.e. enhancing the evaluator sample, might reduce uncertainty and render the trends into significant effects.

Table S 5-2 Modelling evaluator scores with minimum GoF as one predictor: Estimates with 95% confidence intervals of model parameters, below model performance indicators. For information on abbreviations see Table S 5-1.

	Estimate	2.5 %	97.5 %
Intercept[1]	-0.168	-1.170	0.784
Intercept[2]	2.14	1.13	3.13
Intercept[3]	4.37	3.32	5.43
Intercept[4]	6.23	5.11	7.39
Intercept[5]	8.13	6.96	9.35
Minimum_GoF	9.13	7.94	10.50
img_typevalidation	0.806	0.235	1.377
sectorAuthority	-0.32	-1.83	1.12
sectorCRO	0.149	-0.906	1.183
sectorIndustry	-0.148	-1.071	0.772
sectorNotspecified	-0.976	-3.770	1.806
sectorOther	-0.945	-2.299	0.415
Minimum_GoF × img_typevalidation	-2.21	-3.29	-1.11
Experience (momod)	0.0631	-0.2914	0.4444
Experience (momod) × Minimum_GoF	-0.3871	-0.8574	0.0104
sd_evaluator__Intercept	1.272	0.992	1.616
Simo_momodExperience1[1]	0.1839	0.0069	0.6663
simo_momodExperience1[2]	0.2047	0.0082	0.7151
simo_momodExperience1[3]	0.16751	0.00486	0.69148
simo_momodExperience1[4]	0.2616	0.0116	0.7628
simo_momodExperience × Minimum_GoF1[1]	0.2689	0.0134	0.7006

	Estimate	2.5 %	97.5 %
simo_momodExperience × Minimum_GoF1[2]	0.1407	0.0053	0.5751
simo_momodExperience × Minimum_GoF1[3]	0.19640	0.00829	0.65602
simo_momodExperience × Minimum_GoF1[4]	0.2688	0.0125	0.7026
Num.Obs.	640		
ELPD	-812.2		
ELPD s.e.	17.5		
LOOIC	1624.3		
LOOIC s.e.	35.0		

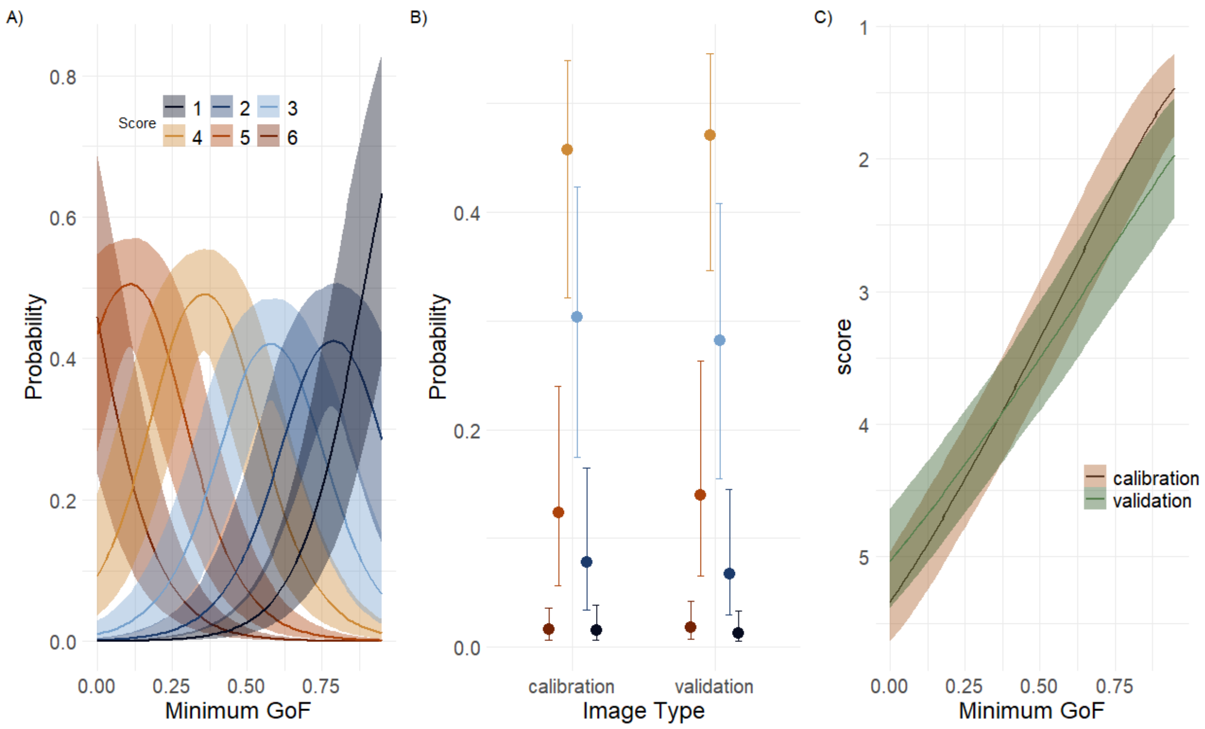

Figure S 5-1 Predictions by the statistical model including all predictors. A) Probability of an image receiving a given visual assessment score (red colors: scores indicating non-acceptance, 4-6; blue colors: scores indicating acceptance, 1-3) depending on Minimum Goodness-of-Fit (GoF). B) Effects of Image Type on predicted visual assessment score values. C) Effects of the interactions between Minimum GoF and Image Type on predicted visual assessment score values (for easier visualization, scores are handled as continuous instead of ordinal variables). The prediction lines and 95% CI are generated from the regression curve at each value of Minimum GoF (all panels) and image type (panel B and C). Other predictors are kept at their reference values and random effects are excluded (for more information see the help page on function 'conditional_effects' in the brms R package).

Influence of modelling experience

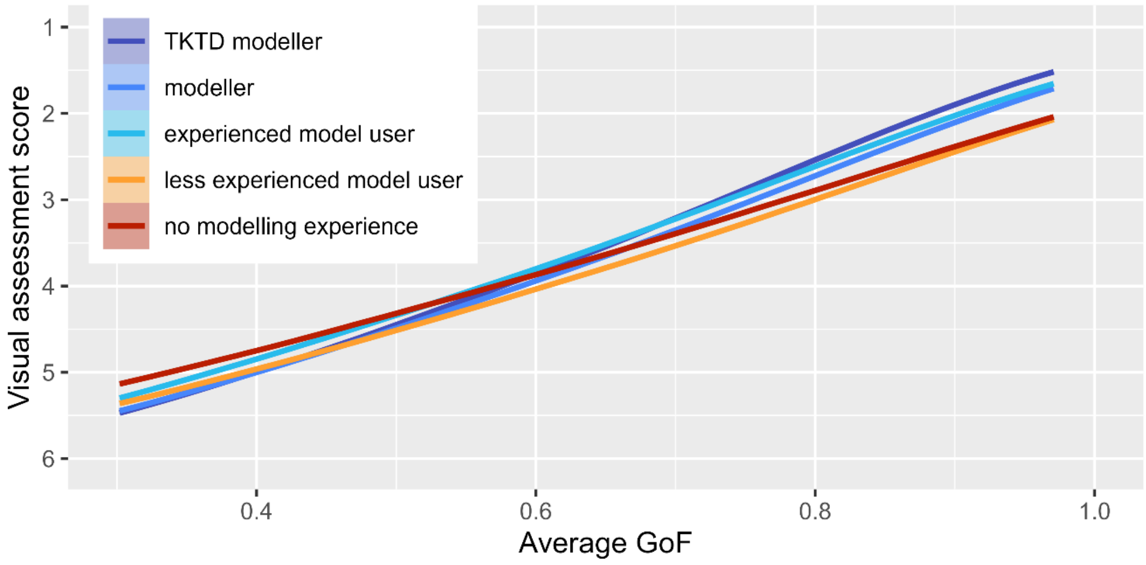

Figure S 5-2 Predictions by the statistical model including all predictors. Effects on predicted visual assessment scores of the interactions between average GoF and modelling experience. For easier visualization, scores are handled as continuous instead of ordinal variables. The prediction lines are generated from the regression curve for the indicated predictors, while other predictors are kept at their reference values (see Table S5-1). Random effects are excluded. For more information see

the help page on function 'conditional_effects' in the brms R package (Bürkner 2018). This figure is a replicate of Fig. 2B in the main manuscript without showing confidence bands.

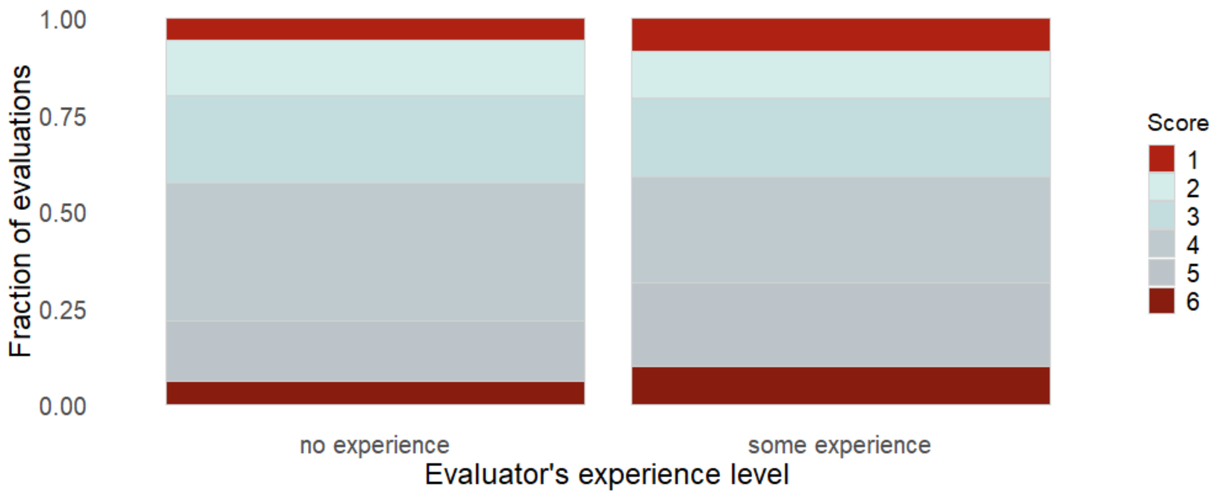

Figure S 5-3 Fraction of evaluations with a given score for evaluators who reported no experience with models (left) or with any other experience level (right). Extreme scores (1: excellent, 6: no resemblance, highlighted in red) are less often chosen by unexperienced evaluators than by experienced ones.

Evaluator acceptance

Table S 5-3 Modelling evaluator acceptance: Estimates with 95% confidence intervals of model parameters, below model performance indicators. For information on abbreviations see Table S 5-1.

	Est.	2.5 %	97.5 %
Intercept	-10.23	-13.54	-7.41
average_GoF	16.0	12.1	20.6
img_typevalidation	-0.771	-3.740	2.000
sectorAuthority	-1.218	-3.174	0.886
sectorCRO	-0.44	-1.80	0.95
sectorIndustry	-0.729	-1.931	0.494
sectorNotspecified	-2.06	-5.80	1.41
sectorOther	-1.718	-3.503	0.121
average_GoF × img_typevalidation	0.0115	-3.9287	4.0963
Experience (momod)	0.31	-0.88	1.40
Experience (momod) × average_GoF	-0.667	-2.105	0.950
sd_evaluator__Intercept	1.442	0.974	2.011
simo_momodExperience1[1]	0.18796	0.00745	0.62902

	Est.	2.5 %	97.5 %
simo_momodExperience1[2]	0.2172	0.0109	0.6638
simo_momodExperience1[3]	0.17253	0.00586	0.72166
simo_momodExperience1[4]	0.2680	0.0124	0.7407
simo_momodExperience × average_GoF1[1]	0.2285	0.0129	0.6396
simo_momodExperience × average_GoF1[2]	0.1341	0.0046	0.5950
simo_momodExperience × average_GoF1[3]	0.3451	0.0137	0.7597
simo_momodExperience × average_GoF1[4]	0.17885	0.00691	0.62095
Num.Obs.	640		
Expected log pointwise predictive density for a new dataset (ELPD)	-233.5		
Standard error (ELPD s.e.)	15.6		
Leave-one-out information criterion (LOOIC)	467.0		
Standard error (LOOIC s.e.)	31.1		

S6. Examples

All figures shown in this chapter are examples of GUTS model predictions shown with observed survival data that were included for evaluation in the survey. Each panel shows survival probability over time for one treatment. Black dots and the corresponding error bars are observations with Wilson intervals. Orange lines are model predictions with credible intervals in grey around them. All model predictions within one figure were made by the same model. Concentration data is intentionally not shown. Panel numbers are internal treatment IDs used by the model and are not relevant.

S6.1. Consistency across evaluators and GoF

High average GoF (0.97), acceptance by all five evaluators - scores 1, 1, 2, 3, 3.

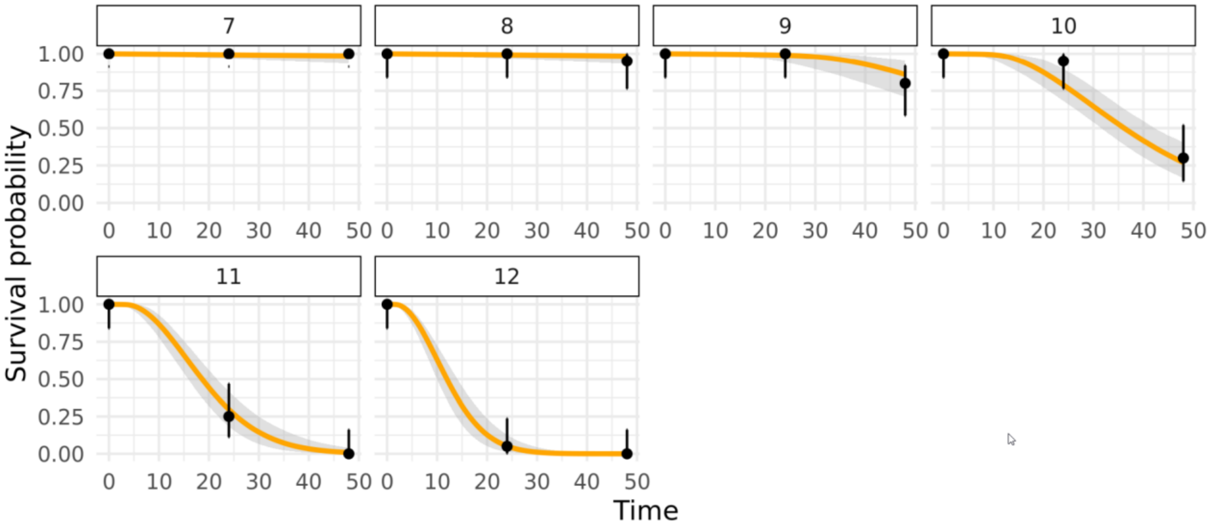

Low average GoF (0.28), rejection by all three evaluators - scores 4 (no reason given), 5, 6 (reason: survival at end not adequately captured in multiple panels).

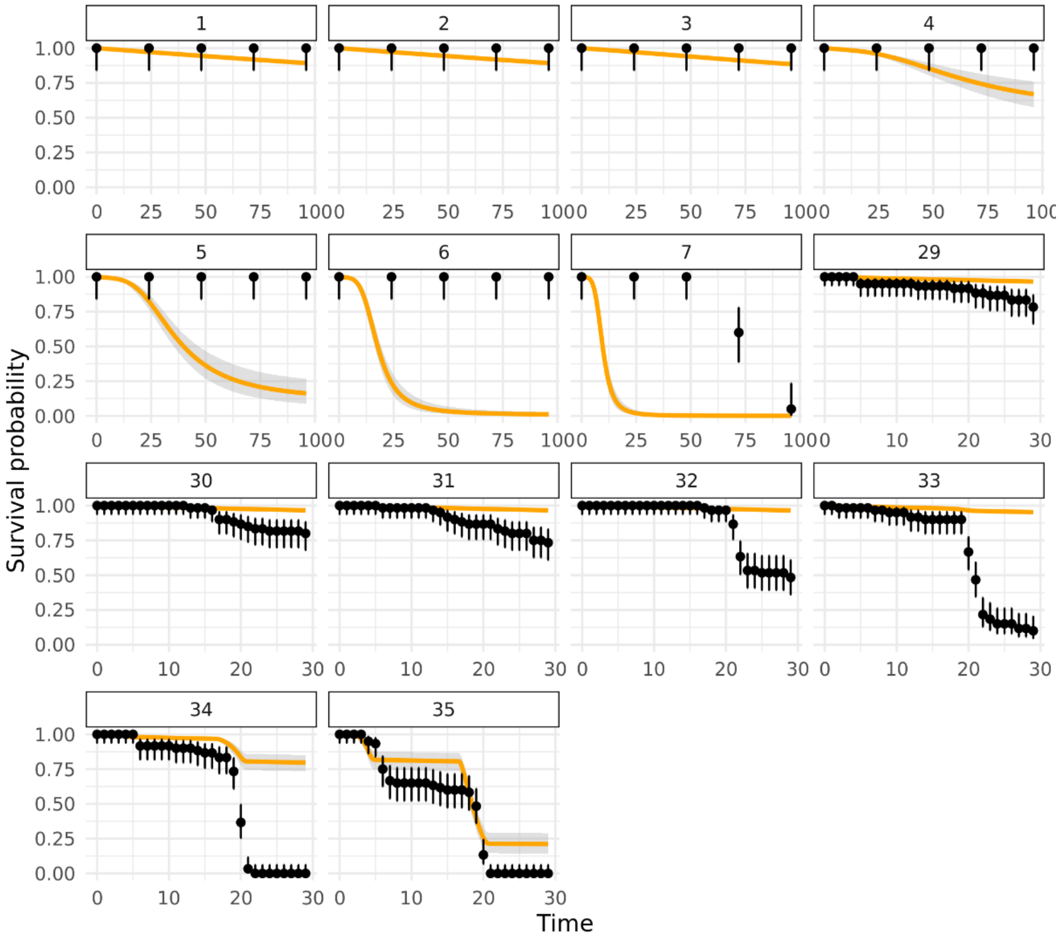

S6.2. Contradiction between evaluators and GoF

Contradiction between a high average GoF (0.83) and consistently bad ratings: 4 (reason: end of survival in one panel not adequately captured), 5, 5 (time course of survival in multiple treatments not adequately captured).

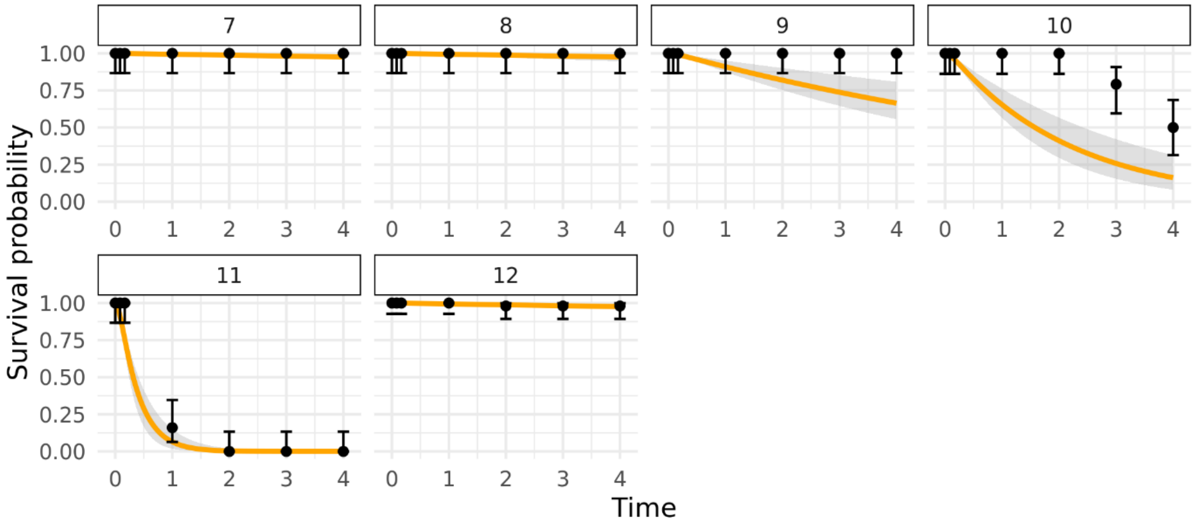

Contradiction between a high average GoF (0.84) and ratings on the border of acceptability: 3, 3, 4 (reason: time course in one treatment not adequately captured), 4 (reason: credible interval too wide).

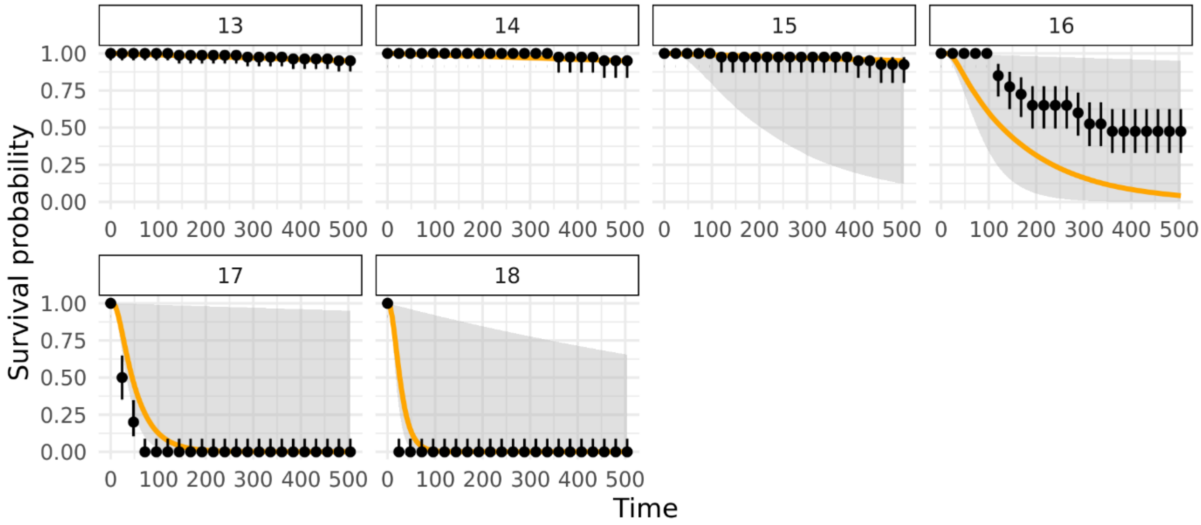

S6.1. Variable scores across evaluators for intermediate GoF

Average GoF in the intermediate range (0.56) and scores are completely variable: 1, 3 and 5 (reason: survival at the end not adequately captured in multiple panels).

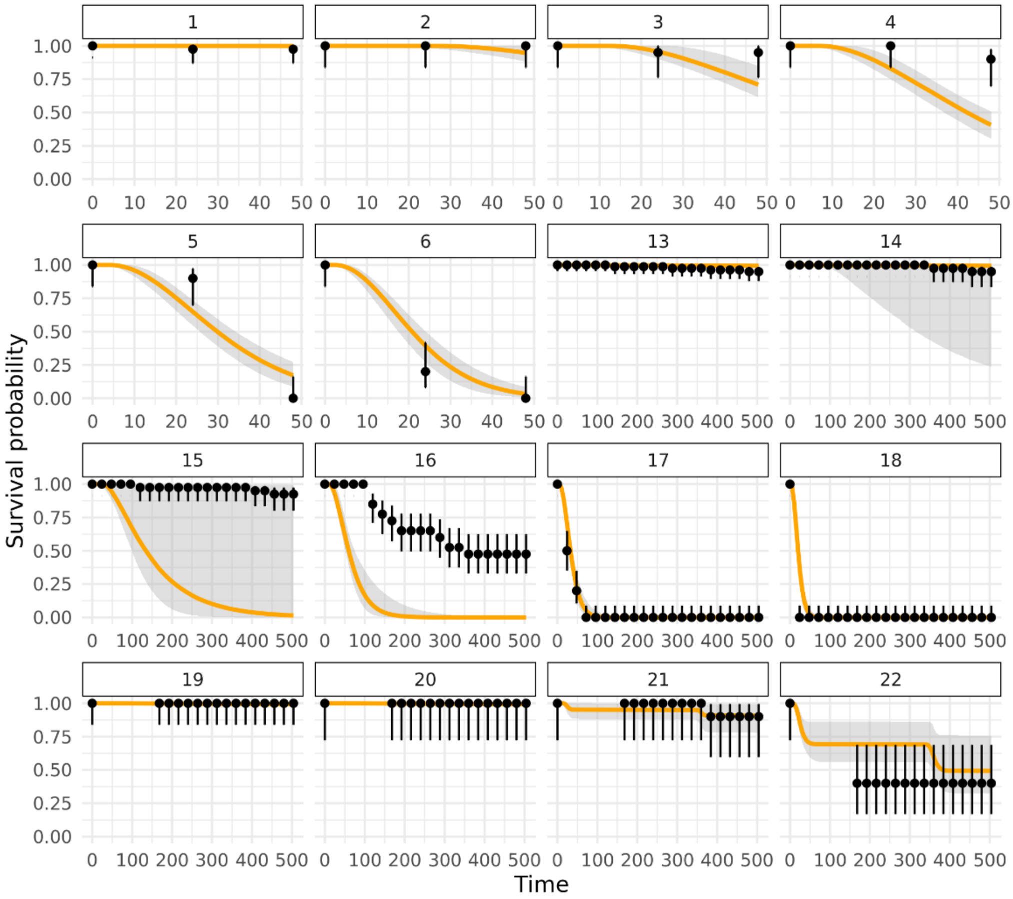

S7. Metric thresholds for model acceptance

Thresholds were predicted as the conditional effect of a metric in the model ACCEPTANCE ~ METRIC. Acceptance was modelled as a binary response (Bernoulli family). Modelling was conducted with R-package brms (Bürkner 2017).

Table S 7-1 Predicted thresholds to distinguish model acceptance from non-acceptance.

GoF	estimate	Lower 95%-C	Upper 95%-CI
NRMSE	20.5	18.6	22.1
PPC	81.7	79.3	84.2
$SPPE_{max}$	8.45	0.571	14.8
$SPPE_{min}$	-35.5	-39.1	-31.8
Average GoF	0.735	0.715	0.755
Minimum GoF	0.540	0.509	0.572

S8. Comparison of scoring DRC and time series fits

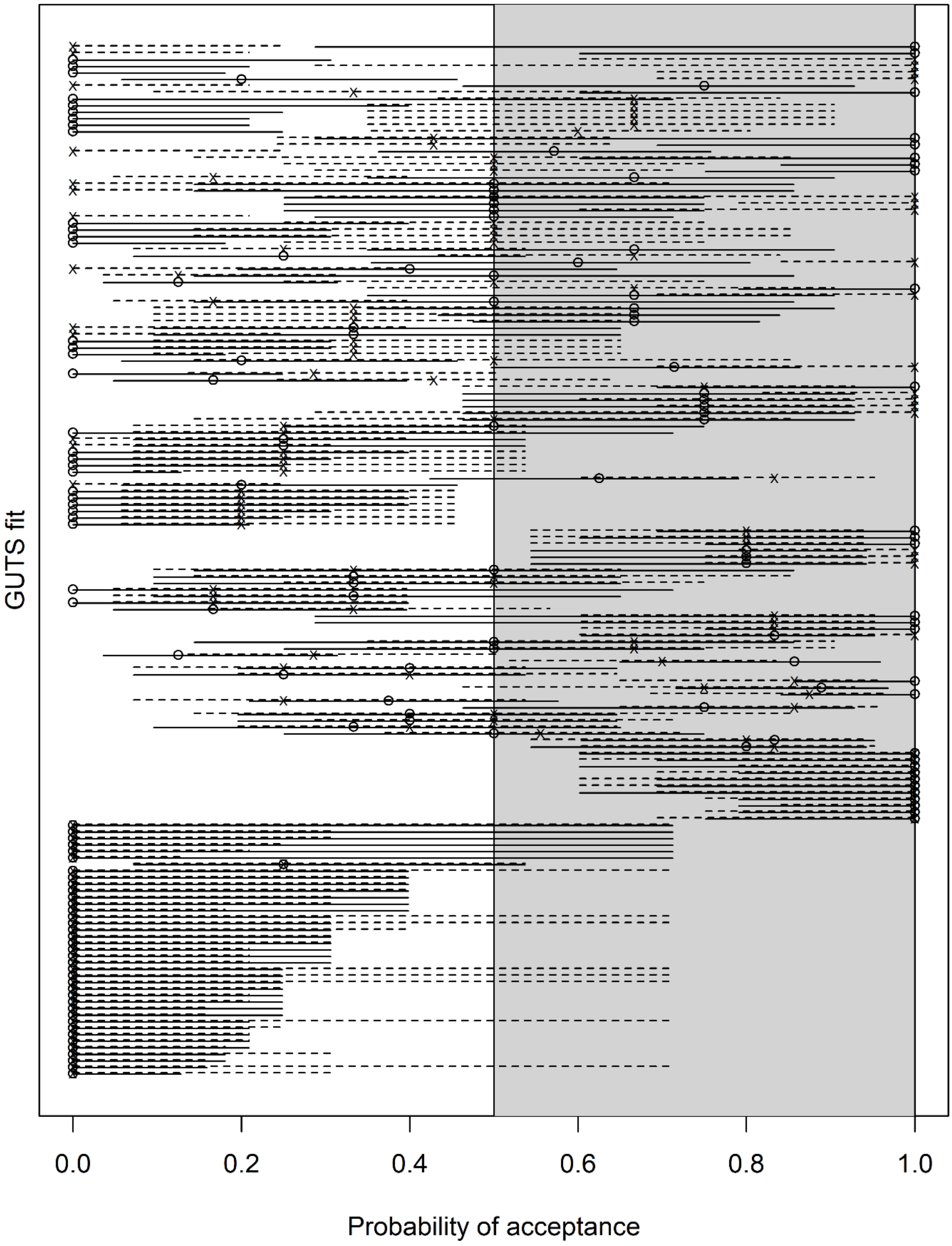

Figure S 8-1 Acceptance probability calculated for each GUTS fit image from the evaluators that rated the images. For each image there are evaluations for DRC (cross, dashed line) and time series (circles, solid lines). Lines reflect the 50% Wilson CIs (with 90% they would range almost across the entire image). The grey range indicates a probability of acceptance larger than 50%. For 24 images, DRC and TS CIs are comprised in the grey area, for one image TS CI >= 0.5 but DRC CI < 0.5, for 3 images, it is vice versa, and for 32 both CIs < 0.5. For the remaining 98 images the CIs overlap the 50% threshold. These numbers could be displayed as the green barplot above. But they are probably not very intuitive? And how to justify the arbitrary 50% CI?

S9. Literature

Ashauer R, Thorbek P, Warinton JS, Wheeler JR, Maund S. 2013. A method to predict and understand fish survival under dynamic chemical stress using standard ecotoxicity data. Environ Toxicol Chem. 32(4):954–965. doi:10.1002/etc.2144.

Baudrot V, Charles S. 2021. TKTDsimulation.jl and tktdjl2r: innovative packages for High Performance Computing of survival predictions in support of environmental risk assessment under time-variable scenarios | bioRxiv. [accessed 2021 Apr 13]. https://www.biorxiv.org/content/10.1101/2021.02.18.431769v1.

Bürkner P-C. 2017. brms: An R Package for Bayesian Multilevel Models Using Stan. J Stat Softw. 80:1–28. doi:10.18637/jss.v080.i01.

Bürkner P-C, Charpentier E. 2020. Modelling monotonic effects of ordinal predictors in Bayesian regression models. Br J Math Stat Psychol. 73(3):420–451. doi:10.1111/bmsp.12195.

Bürkner P-C, Vuorre M. 2019. Ordinal Regression Models in Psychology: A Tutorial. Adv Methods Pract Psychol Sci. 2(1):77–101. doi:10.1177/2515245918823199.

Vehtari A, Gelman A, Gabry J. 2017. Practical Bayesian model evaluation using leave-one-out cross-validation and WAIC. Stat Comput. 27(5):1413–1432. doi:10.1007/s11222-016-9696-4.
